# An Analysis of Natural Variation Reveals That *OsFLA2* Controls Flag Leaf Angle in Rice (*Oryza sativa* L.)

**DOI:** 10.3389/fpls.2022.906912

**Published:** 2022-06-23

**Authors:** Jianhua Jiang, Yuanqing Zhang, Yulong Li, Changmin Hu, Lei Xu, Ying Zhang, Dezheng Wang, Delin Hong, Xiaojing Dang

**Affiliations:** ^1^Institute of Rice Research, Anhui Academy of Agricultural Sciences, Hefei, China; ^2^State Key Laboratory of Crop Genetics and Germplasm Enhancement, Nanjing Agricultural University, Nanjing, China; ^3^Institute of Crop Research, Anhui Academy of Agricultural Sciences, Hefei, China

**Keywords:** genome-wide association mapping, hybrid seed production, natural variation, OsFLA2, rice

## Abstract

Flag leaf angle (FLA) is an important outcrossing trait affecting the hybrid seed production in rice (*Oryza sativa* L.). Natural variation of FLA has been reported in rice, but the molecular basis for this variation is largely unknown. In this study, we investigated the phenotypic values of FLA in 353 rice natural accessions in six environments, which indicated that there was abundant phenotypic variation. We performed a genome-wide association study on FLA using 1.3 million single nucleotide polymorphisms (SNPs). A total of six quantitative trait loci (QTLs) were identified significantly associated with FLA, of which five were located in previously reported QTLs/genes and one was novel. We identified two causal gene loci for FLA, namely, *OsFLA6* and *OsFLA2*; *OsFLA6* was co-localized with the gene *OsLIC*. In addition, the accessions with large and small FLA values have corresponding high and low *OsFLA6* expressions. *OsFLA*2^TT^ allele could increase significantly the seed setting percentage in hybrid F_1_ seed production by field experiment. We also confirmed that the allele *OsFLA2*^*TT*^ increased the FLA compared with that of the isogenic line carrying allele *OsFLA2*^*CC*^ by transgenic complementation experiment. The allele frequencies of *OsFLA6*^*GG*^ *and OsFLA2*^*TT*^ decreased gradually with an increase in latitude in the Northern Hemisphere. Our results should facilitate the improvement of FLA of parents of hybrid rice.

## Introduction

Asian cultivated rice (*Oryza sativa* L.) is one of the most important staple foods feeding more than 3.5 billion people worldwide (GRiSP, 2013). With the human population increasing and the arable land decreasing, increasing the rice grain yield per unit area per unit time is an inevitable choice. The utilization of heterosis is one of the effective strategies to enhance rice grain yield (Yuan, 1997). However, it entails the yearly production of F_1_ hybrid seeds. For hybrid seed production in *O. sativa*, to remove the barriers to cross-pollination at the initial heading stage, farmers usually cut off one-third or one-half of the flag leaf blade of the parents, which not only requires more labor but also high operating skills to avoid injuring the young panicle (Sakamoto et al., 2006; Zhu et al., 2016). In addition, the wound caused by leaf clipping also had an adverse effect on the normal growth of the rice plant. If the flag leaf angle (FLA) in female parents is larger than 90°, leaf clipping can be omitted. Therefore, breeding a male sterile line with larger FLA can not only omit the procedure of flag leaf clipping but also facilitate the mechanization of hybrid rice seed production.

Early research has shown that the FLA trait is controlled by a pair of major genes and a number of minor gene pairs, with a small angle being partially dominant (Shen, 1983). To date, 63 quantitative trait loci (QTLs) for FLA have been identified based on linkage analysis, including 9, 7, 6, 4, 4, 5, 4, 7, 6, 0, 8, and 3 on chromosomes 1–12, respectively (Supplementary Table 1) (Li et al., 1999; Yan et al., 1999; Dong et al., 2003; Kobayashi et al., 2003; Luo et al., 2008; Zhang et al., 2008, 2013; Cai, 2009; Hu et al., 2012; Wang et al., 2012; Bian et al., 2014; Zou et al., 2014; Zhu et al., 2016; Ham et al., 2019). In addition, 100 QTLs for FLA have been detected based on association analysis (Huang et al., 2010; Chen et al., 2012; Lu et al., 2015; Dong H. J. et al., 2018; Dong Z. Y. et al., 2018). Among these QTLs, only one QTL *qFla-8-2* has been fine-mapped and predicted the candidate genes (Zhu et al., 2016). Several genes for leaf angle (refers to all leaves growing on a stem), such as *lla, OsLIC, ILA1*, and *OsARF19*, have been cloned (Wang et al., 2005, 2008; Ning et al., 2011; Zhang et al., 2015, 2016), but no cloned gene responsible for FLA has been reported thus far. Therefore, it is necessary to discover favorable alleles for FLA to enhance the yield of hybrid seed production in rice.

In this study, we performed a genome-wide association study (GWAS) by combining the FLA of 353 rice accessions on six environments with single nucleotide polymorphism (SNP) data and identified significant SNP loci. Furthermore, we identified a novel causative gene *OsFLA2* for FLA using the gene-based association (GBA) method. The function of *OsFLA2*^*TT*^ was validated using the transgenic complementation test. These results filled a gap in gene cloning and functional analysis of FLA characteristics. This study sets the stage for further improvement of the FLA of the parents of hybrid rice.

## Materials and Methods

### Rice Accessions

In our previous study, the three stigma characteristics of 353 accessions were reported by Dang et al. (2020) (Supplementary Table 2). In this study, using the same 353 accessions, we investigated the FLA characteristic following the same field plant and management. These accessions were grown across six different environments, over 3 years (2017–2019) and two locations of Nanjing (32°07'N, 118°64'E) and Hefei (31°52'N, 117°17'E).

### Phenotypic Investigation

By following the method reported by Zhu et al. (2016), the FLA of the plants was measured with a protractor at the stage of the panicle on the main stem heading 10 cm above the flag leaf lamina joint. For each accession, the average FLA value of 10 plants was used as the phenotypic value.

### Genome-Wide Association Study

The sequences for the accessions are available at EBI European Nucleotide Archive with the accession number ERP000106 and NCBI Sequence Read Archive with the accession numbers PRJNA171289 and PRJNA554986 (Huang et al., 2012; Chen et al., 2014; Dang et al., 2020). According to the method reported by Li and Durbin (2010), we used the Bowtie 2 software to align the reads to Os-Nipponbare-Reference-IRGSP 1.0. A total of 95% of reads were mapped to the Nipponbare genome with a mapping score >60. Then, we used the HaplotypeCaller of GATK 3.8-0 to perform the SNP calling. We used the Beagle software version 4.1 (Browning and Browning, 2009) to impute the missing data in genotype data. Finally, we obtained 1,326,094 SNPs with a minor frequency (MAF) >5% and a missing rate <18% and used them to estimate kinship coefficients and for GWAS. We used the TASSEL 5.2.1 software (Bradbury et al., 2007) to calculate the kinship matrix. Based on the mixed linear model (MLM), the GWAS was conducted using the R package Genomic Association and Prediction Integrated Tool (Lipka et al., 2012). According to the methods reported by Yang et al. (2014), if the association loci exceeded the *P*-value thresholds (<1 × 10^−7^) with clear peak-like signals (≥3 significant SNPs) and were in the 200-kb region of the leading SNP, we considered the region as a QTL. We used the R package “LDheatmap” to construct the linkage disequilibrium (LD) heatmaps surrounding peaks in the GWAS (Shin et al., 2006). The Manhattan and quantile-quantile plots were drawn using the R package qqman (Turner, 2014). We used the correction method of Benjamini and Hochberg (1995) to calculate the false discovery rate (FDR) and determine the genome-wide significance thresholds of the GWAS, which was 1.0 × 10^−7^ at a nominal level of 0.05. The position of SNPs in *OsFLA2* and *OsFLA6* is based on data from the MSU Rice Genome Annotation Project (http://rice.plantbiology.msu.edu).

For GBA analysis, we used the MLM following the equation: *y* = *x*β + *zu* + *e*. Where *y* represents a vector of phenotype; *x* represents a matrix of fixed effects such as gene haplotype; β represents a vector of effects; *z* represents an incidence matrix relating *y* to *u*; and *e* represents a matrix of residual effects. First, based on polymorphisms localized on the coding regions, we performed gene haplotype identification using an R script. The difference in haplotype was used as a dummy variable, which was used as fixed effects using the R package “rrBLUP” version 4.3 (Endelman, 2011).

### RNA Extraction and qRT-PCR

The total RNA was extracted from flag leaf and flag leaf lamina joint at development stages 5–8 [as per the criterion described by Itoh et al. (2005)] using the ultrapure RNA kit (OMEGA BIO-TEK, https://www.omegabiotek.com), sampled from the six accessions (three accessions with smaller FLA and three accessions with larger FLA). The RNase-free DNase I treatment (Vazyme, http://www.vazyme.com) was used to remove any genomic DNA contamination. In addition, the HiScript II Q RT SuperMix (Vazyme, http://www.vazyme.com) was used to perform the first-strand cDNA synthesis by reverse transcription from 1 μg of RNA. We used the 18S rRNA gene as an internal control. We performed the qRT-PCR in a 96-well thermocycler (Roche Applied Science LightCycler 480) using SYBR Green (Vazyme, http://www.vazyme.com) and set the cycling conditions as follows: first, denaturation (95°C, 5 min); second, amplification and quantification program-40 cycles (95°C for 10 s, 60°C for 30 s, and 72°C for 60 s) with a single fluorescence measurement; third, the melting curve (60–95°C) with a heating rate of 0.1°C per second and continuous fluorescence measurement; and finally, cooling step (40°C). We performed the three independent replicates. The primer sequences of qRT-PCR are shown in Table 1. We calculated the relative gene expression of the target gene using the following equation (Pfaffl, 2001): *Exp*=2^−Δ*Ct*^, where ΔCt =Ct_target gene_ - Ct_18SrRNA_.

**Table 1:** Primers used in this study.

**Primer name**	**Forward primers (5^**′**^-3^**′**^)**	**Reverse primers (5^**′**^-3^**′**^)**
Primers for quantitative real-time RT-PCR:
RT1-18S	GAGATGGGTAGGGACGTGGAT	TGGTACGTCTCGTCCACCTT
RT2-*Os02g0142875*	GGAGGCTTGCGGTGAGTATC	AAGCAGGCGTTACATTCCCC
RT3-*Os06g0704300*	GCAGTTCATCGGAGAAGCGA	CGGGAATGAAGTGAGTACCGA
**Primers for plasmid construction:**
c*OsFLA2*	cagtCACCTGCaaaacaactgggaaacagcagatggaattc	cagtCACCTGCaaaatacactagttcttggcttcttgcaca
Primers for genotype identification of gene *OsFLA2*:
	GGTTGTGGCTTCCTAGGTCC	ATGACCAACCAACTGGGCAA

### Generation of *OsFLA2* Transgenic Plants

The full-length genomic DNA of *Os02g0142875* was amplified by PCR from A7444 rice and cloned into the pBWA(V)HII vector (Table 1). This construct (pBWA(V)HII-*OsFLA2*) was then transformed into Nipponbare and 7001S by *Agrobacterium* EHA105, respectively. Additionally, the corresponding empty vector transformed into Nipponbare was used as a control. Thirty-two independent T_1_ seedlings obtained were grown to maturity under natural conditions. In the next rice-growing season (May to October), the T_2_ seeds harvested from T_1_ plants at the maturity stage were grown in the paddy field. At the tillering stage, the three allele genotypes (TT, TC, and CC) on the *Os02g0142875* locus were determined using the primers listed in Table 1, and the FLA was measured in the *Os02g0142875*^*TT*^ and *Os02g0142875*^*CC*^ plants at full heading stage.

### Evaluation of the Utilization Value for *OsFLA2* Alleles

We selected isogenic lines 7001S^*FLA*2−*CC*^ (small FLA) and 7001S^*FLA*2−*TT*^ (large FLA) as the female parents and variety 9311 as the male parent to perform an actual hybrid F_1_ seed production experiment. 7001S carried the CC allele. 7001S^*FLA*2−*TT*^ obtained in this study is an isogenic line. The male parent and female parent inter-planted and the planting-to-row ratio is 2:6:2. At flowering time, artificial pollination was conducted two times each day. After 30 days, the panicle from the female parents was harvested. The seed setting percentage was calculated to evaluate the potential value of the *OsFLA2* allele in hybrid F_1_ seed production.

## Results

### Phenotypic Statistics of FLA in Natural Rice Accessions

**Figure 1:**
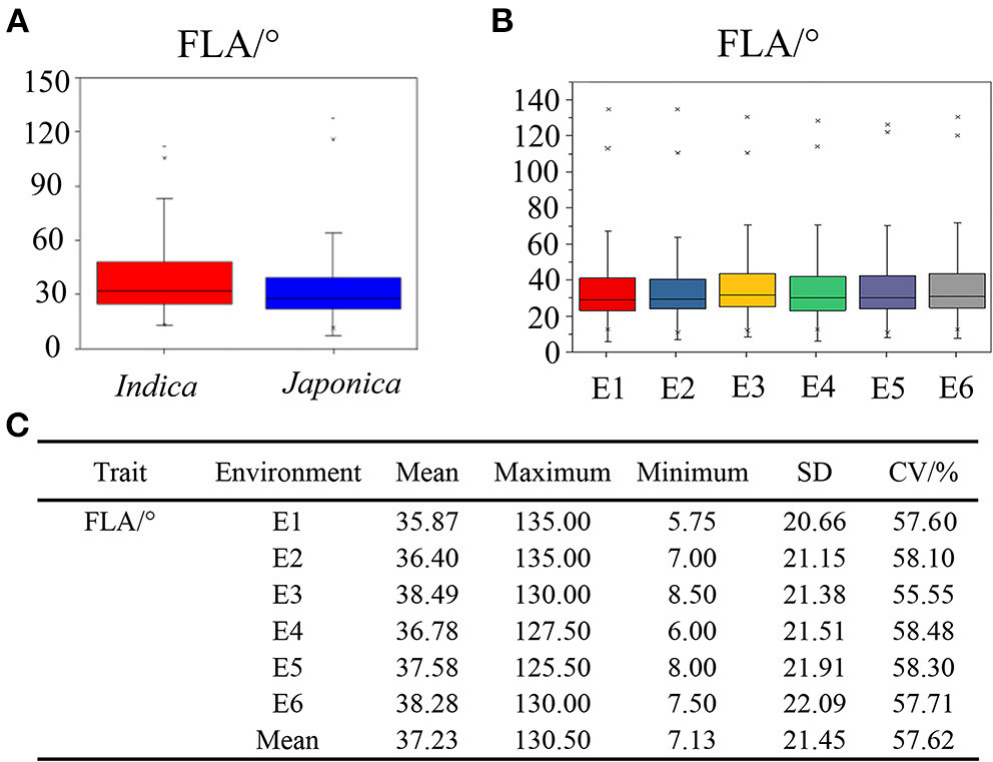
Phenotypic characteristics of flag leaf angle (FLA) in *indica* subgroup, *japonica* subgroup, and six environments, respectively. **(A)** Phenotypic value distributions of FLA in *indica* subgroup and *japonica* subgroup. The number of varieties within each population was 172 and 181, respectively. **(B)** Phenotypic value distributions of FLA in six environments. **(C)** Phenotypic statistics of FLA in six environments. The box edges represent the upper and lower quantiles, with the median value shown by the black line in the middle of the box. Vertical lines represent the data from the lowest quantile to the top quantile. Individuals falling outside the range of the whiskers are shown as asterisks. E1, Nanjing in 2017; E2, Nanjing in 2018; E3, Nanjing in 2019; E4, Hefei in 2017; E5, Hefei in 2018; E6, Hefei in 2019.

The phenotypic value of FLA was investigated in the 353 rice accessions containing *indica* and *japonica* subspecies across six environments. The distributions of average value over the six environments for FLA in *indica* and *japonica* subspecies are shown in Figure 1A. Compared with *indica* rice, the *japonica* rice population had lower values for FLA (Figure 1A). In the 353 accessions, the mean value of FLA was calculated per environment, ranging from 35.87 ± 20.66° to 38.49 ± 21.38°, with coefficients of variation (CV) across the six environments from 55.55 to 58.48% (Figures 1B,C). These results showed that there existed abundant phenotype variation in the population studied. The scatterplots of FLA across different years and locations were carried out and shown in Supplementary Figure 1, indicating that FLA was genetically stable in six environments. Based on the results of the joint analysis of variance for FLA, we found that there were significant differences among genotypes, no significant differences among the environments, and significant differences in the interactions of genotypes with environments (Supplementary Table 3). These results indicated that although the environment had an effect on FLA, the abundant phenotypic variation of FLA was mainly attributable to variation in genotype.

### Genome-Wide Association Mapping for FLA

**Figure 2:**
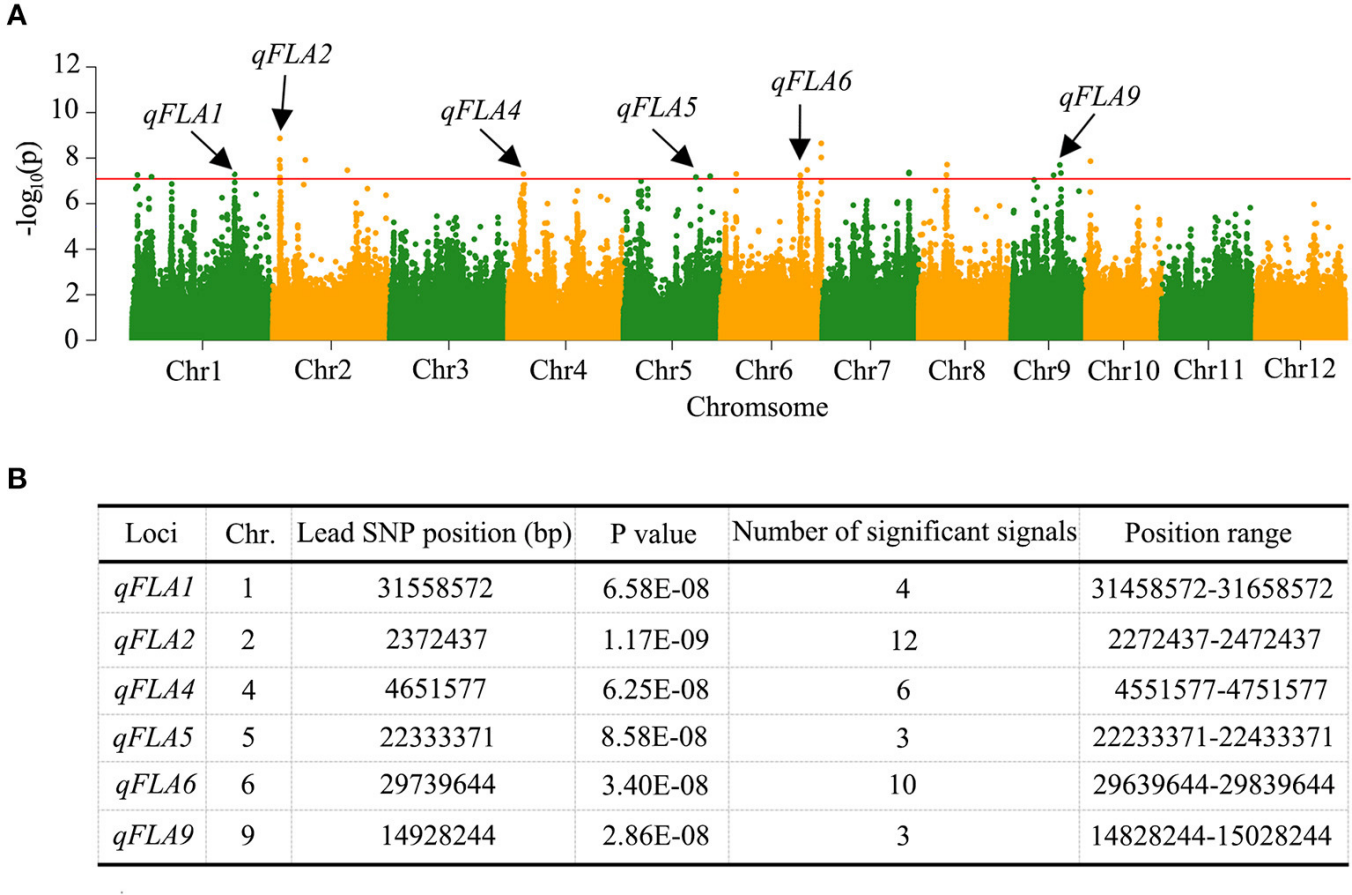
Quantitative trait locus (QTL) identification for FLA by genome-wide association study (GWAS) in rice. **(A)** Manhattan plots for the whole population of rice accessions. The vertical axis indicates the negative log_10_-transformed *P*-values. The dots above the red line mean the significant single nucleotide polymorphisms (SNPs) in the QTL region. The black arrows indicate the QTL identified. **(B)** Information of the identified QTL.

Based on the mixed linear model with correction of kinship bias, GWAS on FLA trait of 353 accessions with high-quality SNPs (MAF > 0.05) was carried out. The significant SNP associations with FLA were illustrated in Manhattan plots (Figure 2A). We considered the region as a QTL when more than three significant SNPs exceeding the threshold value of 1 × 10^−7^ were in the 200-kb interval of the leading SNP. According to this criterion, we identified six QTLs associated with FLA (Figure 2B). Among them, two QTLs, namely, *qFLA2* and *qFLA6*, own a relatively large number of significant SNPs 12 and 10, respectively. We considered *qFLA2* and *qFLA6* as the major QTLs for FLA to analyze further.

### Allele *OsFLA6^*GG*^* Increases FLA

**Figure 3:**
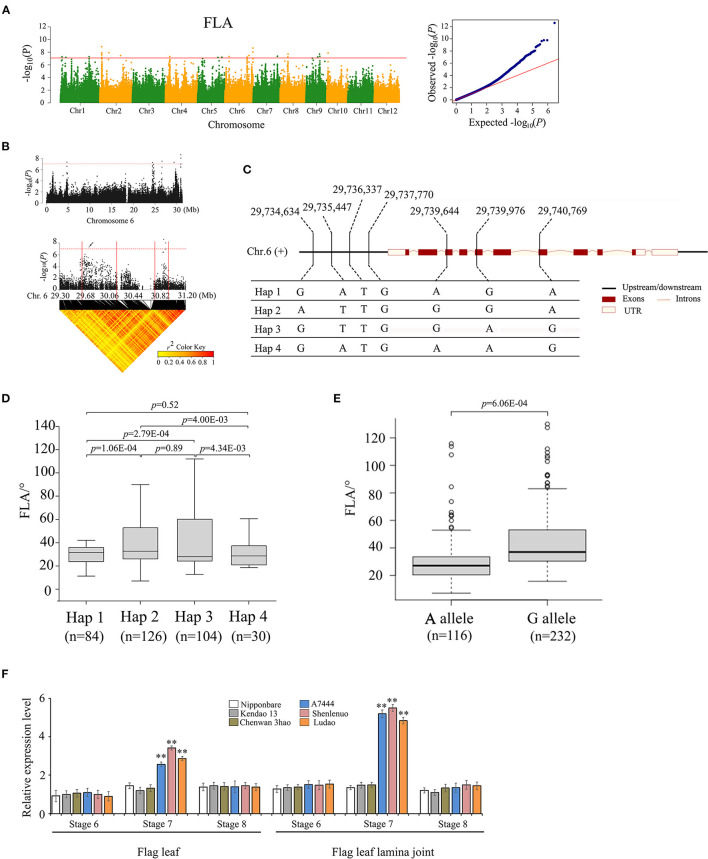
Genome-wide association study for FLA and identification of the candidate gene *OsFLA6* (*Os06g0704300*). **(A)** Manhattan plots for FLA. Red line means the significance threshold (–log_10_ *P* = 7.0). **(B)** Local Manhattan plot (top) and linkage disequilibrium (LD) heatmap (bottom) on chromosome 6. The arrow indicates the position of nucleotide variation in *Os06g0704300*. The candidate region lies between the red solid lines. **(C)** Haplotypes of *OsFLA6* associated with flag leaf angle in rice. **(D,E)** Box plots of flag leaf angle in accessions containing the different haplotypes **(D)** and elite alleles **(E)**. Center line, median; box limits, upper and lower quartiles; whiskers, 1.5 × the interquartile range; dots, outliers. Differences between the alleles were statistically analyzed based on Welch's *t*-test. **(F)** Relative expression of *Os06g0704300* in flag leaf and flag leaf lamina joint at development stages 6–8 from the three accessions (i.e., Ludao, Shenlenuo, and A7444) with a large FLA and the three accessions (i.e., Nipponbare, Kendao 13, and Chenwan 3hao) with a small FLA, determined by qRT-PCR (**P < 0.01, *P < 0.05, two-tailed Welch's *t*-test). Data are presented as means ± SE; *n* = 3 independent biological replicates.

For the association signal in the 29.63–29.83 Mb region on chromosome 6, there were 40 genes for FLA identified (Figures 3A,B). In this region, 32 of 40 genes contain nonsynonymous SNPs (Supplementary Tables 4, 5). Only one nonsynonymous SNP was found to be significantly associated with FLA (–log_10_*P* ≥ 7.0) using two methods of GWAS and GBA (Supplementary Table 4); it was located within the gene locus *Os06g0704300*. Hereafter, gene *Os06g0704300* is referred to as *OsFLA6*. The full length of *OsFLA6* is 3,620 bp, including 11 exons and 10 introns. The SNPs of *OsFLA6* occurred in the 2 kb upstream and the coding sequence of the gene. This resulted in four haplotypes of *OsFLA6* being identified (Figure 3C). The elite haplotypes Hap 2 and Hap 3 were associated with larger FLA while haplotype Hap 1 and Hap 4 were associated with smaller FLA (Figure 3D). Among them, only one SNP locus (29,739,644) was significantly associated with FLA (Supplementary Table 4), which causes a base change from base A to base G at nucleotide (nt) 328 in the coding sequence, resulting in an amino acid change from threonine (T) to alanine (A) at amino acid 110. The average FLA value of 116 accessions carrying the allele *OsFLA6*^*AA*^ was 26.5 ± 11.8°. The average FLA value of 232 accessions carrying the allele *OsFLA6*^*GG*^ was 37.5 ± 12.3°. The differences in FLA value between alleles *OsFLA6*^*AA*^ and *OsFLA6*^*GG*^ were highly significant (Welch's *t*-test; *P* = 6.06E−04) (Figure 3E).

Furthermore, we performed quantitative real-time polymerase chain reaction (qRT-PCR) analysis of flag leaf and flag leaf lamina joint at differentiation stages 6, 7, and 8, respectively, sampling from three accessions (i.e., A7444, Shenlenuo, and Ludao) with large FLA and three accessions (i.e., Nipponbare, Kendao 13, and Chenwan 3hao) with small FLA. The results showed that the expression of *OsFLA6*^*GG*^ was higher than that of *OsFLA6*^*AA*^ in flag leaf and flag leaf lamina joint at differentiation stage 7, but no significant difference was found at stages 6 and 8 (Figure 3F). We also found that the expression of *OsFLA6*^*GG*^ in each of the three accessions with larger FLA was significantly higher than that of *OsFLA6*^*AA*^ in each of the three accessions with smaller FLA (Figure 3F). Based on the website of China Rice Data Center (http://www.ricedata.cn/gene/list/286.htm), we found that the gene locus *Os06g0704300* was identical to *OsLIC* (*O. sativa* leaf and tiller angle increased controller), which encodes a CCCH-type zinc finger protein and regulates leaf angle and tiller angle through the BR signaling pathway (Wang et al., 2008; Zhang et al., 2012). Research on the function of *LIC* has been reported, and we will no longer study the function of *OsFLA6*^*GG*^.

### Introduction of the Allele *OsFLA2^*TT*^* Increases FLA

**Figure 4:**
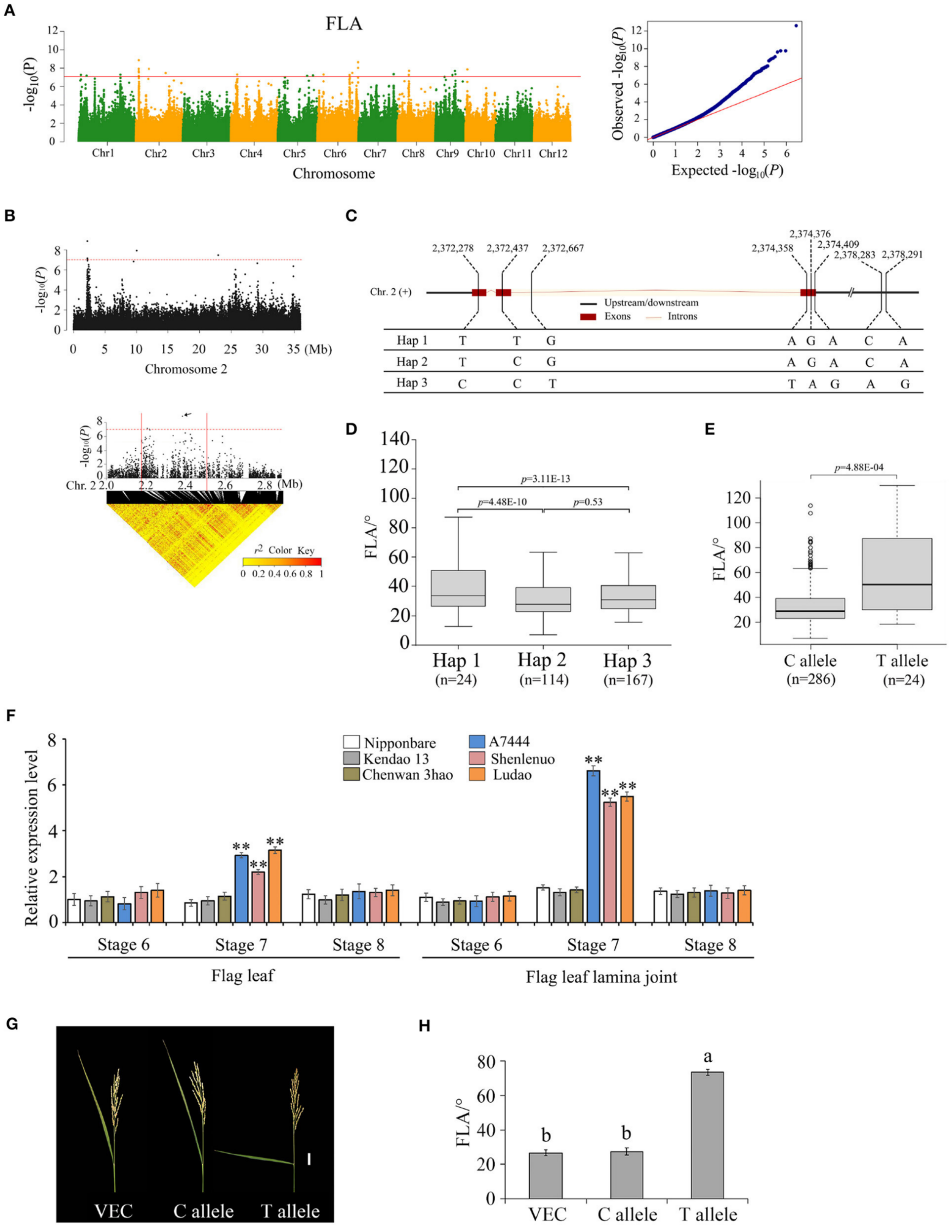
Genome-wide association study for FLA and identification of the causal gene *OsFLA2* (*Os02g0142875*). **(A)** Manhattan plots for FLA. Red line indicates the significance threshold (–log_10_ *P* = 7.0). **(B)** Local Manhattan plot (top) and LD heatmap (bottom) on chromosome 2. The arrow indicates the position of nucleotide variation in *Os02g0142875*. The candidate region lies between the red solid lines. **(C)** Haplotypes of *OsFLA2* associated with flag leaf angle in rice. **(D,E)** Box plots of flag leaf angle in accessions containing the different haplotypes **(D)** and elite alleles **(E)**. Center line, median; box limits, upper and lower quartiles; whiskers, 1.5 × the interquartile range; dots, outliers. Differences between the alleles were statistically analyzed based on Welch's *t*-test. **(F)** Relative expression of *Os02g0142875* in flag leaf and flag leaf lamina joint at development stages 6–8 from the three accessions (i.e., Ludao, Haomake, and A7444) with a large FLA and the three accessions (i.e., Nipponbare, Kendao 13, and Chenwan 3hao) with a small FLA, determined by qRT-PCR (**P < 0.01, two-tailed Welch's *t*-test). Data are presented as means ± SE; *n* = 3 independent biological replicates. **(G)** Images of FLA of transgenic plants transformed with the empty vector (VEC), C allele, and T allele. Scale bar = 3 cm. **(H)** FLA of transgenic plants. Data are presented as means ± SE (*n* = 20).

For the association signal in the 2.27–2.47 Mb region on chromosome 2, there were 19 genes for FLA identified (Figures 4A,B). Based on SNP information, 10 of the 19 genes contain nonsynonymous SNPs (Supplementary Tables 6, 7). However, only one nonsynonymous SNP was significantly associated with FLA using two methods of GWAS and GBA (Supplementary Table 6); it was located within the gene locus *Os02g0142875*. Hereafter, gene *Os02g0142875* is referred to as *OsFLA2*. The full length of *OsFLA2* is 2,177 bp, including three exons and two introns. Gene *OsFLA2* encodes a 95 amino acid protein. For *OsFLA2*, there was no putative conserved domain detected. The SNPs of *OsFLA2* occurred in the coding sequence, the intron, and the 2 kb downstream of the gene. This resulted in three haplotypes of *OsFLA2* being identified (Figure 4C). The elite haplotype Hap 1 was associated with larger FLA while haplotypes Hap 2 and Hap 3 were associated with smaller FLA (Figure 4D). For the five exon SNPs, one SNP site (2,372,278 bp) was synonymous, and the other four were nonsynonymous. Among the four nonsynonymous SNPs, only one SNP site (2,372,437) was significantly associated with FLA (Supplementary Table 6). The SNP site (2,372,437) causes a base change from base C to base T at nt 137 in the cDNA sequence, which results in an amino acid change from serine (S) to phenylalanine (F) at amino acid 46. The average FLA values of 286 accessions carrying the allele *OsFLA2*^*CC*^ were 28.5 ± 10.5°. The average FLA values of 24 accessions carrying the allele *OsFLA2*^*TT*^ were 51.5 ± 13.1°. The difference in FLA values between the *OsFLA2*^*CC*^ and *OsFLA2*^*TT*^ genotypes was highly significant (Welch's *t*-test; *P* = 4.88E−04) (Figure 4E).

The qRT-PCR results showed that there were expressions for *OsFLA2*^*TT*^ and *OsFLA2*^*CC*^ in flag leaf and flag leaf lamina joint at differentiation stages 6, 7, and 8 (Figure 4F). The expression of *OsFLA2*^*TT*^ was higher than that of *OsFLA2*^*CC*^ in flag leaf and flag leaf lamina joint at differentiation stage 7, but no significant difference was found at stages 6 and 8 (Figure 4F). We also found that the expression of *OsFLA2*^*TT*^ in each of the three accessions (i.e., A7444, Shenlenuo, and Ludao) with large FLA was significantly higher than that of *OsFLA2*^*CC*^ in each of the three accessions (i.e., Nipponbare, Kendao 13, and Chenwan 3hao) with small FLA (Figure 4F). These results suggested that enhanced expression of *OsFLA2*^*TT*^ might increase FLA.

According to the results of GWAS, no SNP loci located in the promoter region of *OsFLA2* were associated with FLA. Based on the website of promoter functional elements (http://bioinformatics.psb.ugent.be/webtools/plantcare/html/#opennewwindow), we found that there were no SNP loci in the *cis*-element regulatory region. So, we speculated that phenotypic variation between the accessions with the TT allele and those with the CC allele was caused by SNP loci in the coding sequence region. Next, we conducted a transformation of the *OsFLA2* gene to confirm it. The genome sequence of the allele *OsFLA2*^*TT*^ and empty vector were introduced into Nipponbare, respectively. Compared with the plants of the Nipponbare genome, plants transformed with the allele *OsFLA2*^*TT*^ had a larger FLA, whereas those transformed with the empty vector showed no phenotypic change (Figures 4G,H). We investigated the FLA phenotype of plants including Nipponbare-empty vector, Nipponbare-CC allele, and Nipponbare-TT allele, respectively. Twenty plants were investigated for each type. The results showed that the FLA phenotype value between plants with Nipponbare-empty vector and Nipponbare-CC allele has no significant difference. There was a highly significant (*P* < 0.01) difference in FLA phenotype value between plants with the Nipponbare-CC allele and Nipponbare-TT allele. These results showed that *OsFLA2* was the causal gene for FLA on chromosome 2.

### *OsFLA*2^TT^ Can Increase the Seed Setting Percentage in Hybrid F_1_ Seed Production

**Figure 5:**
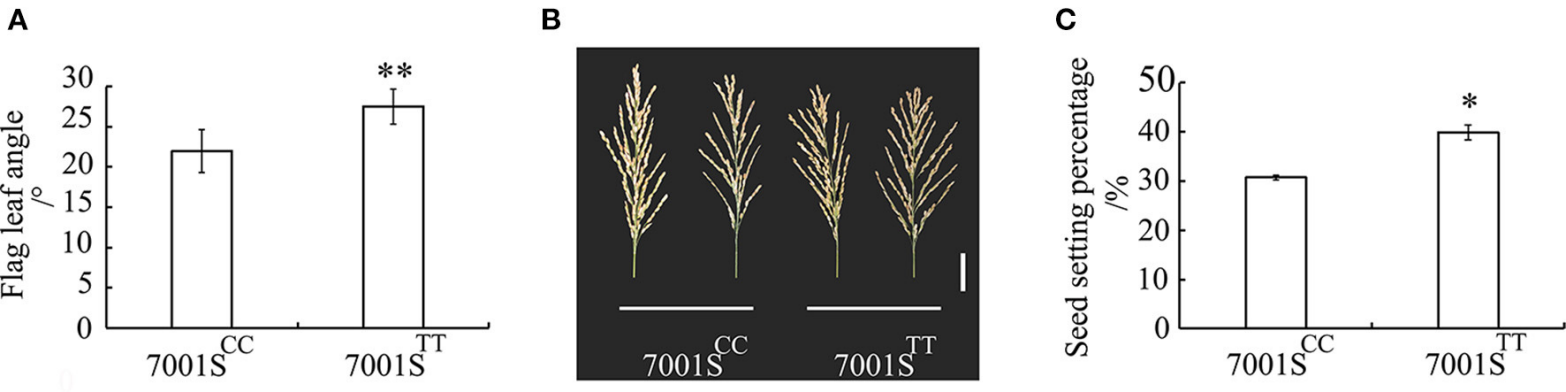
Evaluation of the utilization value for *OsFLA2* alleles. **(A)** Comparison of the FLA of 7001S^*FLA*2−*CC*^ and 7001S^*FLA*2−*TT*^. Data represent means ± SD (*n* = 40 independent plants). **P < 0.01, Student's *t*-test. **(B)** Panicle morphology of 7001S^*FLA*2−*CC*^ and 7001S^*FLA*2−*TT*^. Scale bar, 5 cm. **(C)** Comparison of the seed setting percentage of 7001S^*FLA*2−*CC*^ and 7001S^*FLA*2−*TT*^. Data represent means ± SD (*n* = 32 independent plants). *P < 0.05, Student's *t*-test.

The FLA of 7001S^*FLA*2−*TT*^ was significantly larger than that of 7001S^*FLA*2−*CC*^ (Figure 5A). We investigated the seed setting percentage of the two combinations, 7001S^*FLA*2−*TT*^ × 9311 and 7001S^*FLA*2−*CC*^ × 9311, respectively. The seed setting percentage of 7001S^*FLA*2−*TT*^ was 39.83%, which is significantly (*P* < 0.05) higher than that of 7001S^*FLA*2−*CC*^ (30.72%) (Figures 5B,C). These results show that the *OsFLA*2^TT^ allele could increase significantly (*P* < 0.05) the seed setting percentage in hybrid F_1_ seed production.

### Allele Frequency Distribution of *OsFLA2* and *OsFLA6*

**Figure 6:**
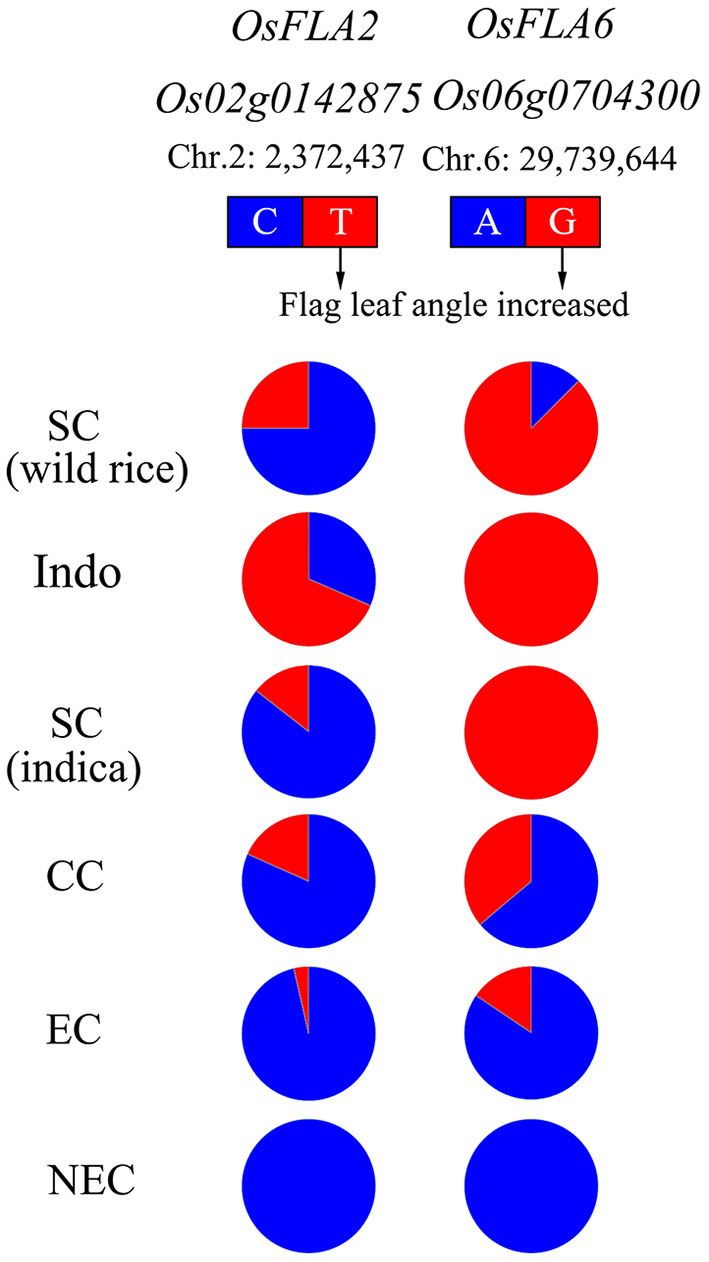
The gene allele frequency differences at the causal polymorphisms of *OsFLA2* and *OsFLA6* in five geographic groups. The type of reference allele is indicated in blue, and the alternative is indicated in red. Indo, Indonesia; SC, southern China; CC, central China; EC, eastern China; NEC, northeastern China. The accessions from SC were mainly wild rice and *indica* subspecies. The accessions from Indo were mainly tropical *japonica* subspecies. The accessions from CC were mainly *indica* subspecies. The accessions from EC and NEC were mainly temperate *japonica* subspecies.

To elucidate the allele types of *OsFLA2* and *OsFLA6* loci in wild rice, we analyzed the sequences of 12 wild rice reported by Dang et al. (2020). The sequencing analysis results showed that the alleles of both *OsFLA2* and *OsFLA6* loci were all found in wild rice (Figure 6). We investigated the regional differentiation of diverse alleles on *OsFLA2* and *OsFLA6* gene loci. The allele *OsFLA2*^*TT*^ (large FLA) is mainly distributed in accessions collected from low-latitude regions, such as southeastern Asia. For *OsFLA2*^*CC*^ (small FLA), we found that it is mainly distributed in accessions collected from high-latitude regions, such as northeastern China, and FLA decreases with the increase of latitude (Figure 6). A similar situation was observed for *OsFLA6*, in which the allele *OsFLA6*^*GG*^ was mainly distributed in accessions collected from southern China and southeastern Asia (Figure 6).

Furthermore, by consulting the published database in rice, we selected 30 wild rice (*Oryza rufipogon*) (https://www.ebi.ac.uk/ena/browser/view/PRJEB2829) and 202 *O. sativa* (http://ricevarmap.ncpgr.cn/two_cultivars_compare/) to analyze the allele frequency distribution of *OsFLA2* and *OsFLA6* (Supplementary Tables 8–11). The results in Supplementary Figure 2 show a similar allele frequency distribution of *OsFLA2* and *OsFLA6* like that in Figure 6 except TRJ. We speculated the reason is that the TRJ in the database was from many sources while the TRJ in this study was only from Indonesia.

## Discussion

In this study, we investigated the FLA phenotype data in 353 rice accessions and confirmed that there existed a rich phenotypic variance. The CV for FLA ranged from 55.55% (E3) to 58.48% (E4) (Figure 1C). The results of a joint variance analysis indicated that the variations in FLA were the main contribution to diverse genotypes, although significant interactions between genotypes and environments were detected. In conclusion, these results provide the basis to mine the elite alleles for FLA.

In this study, we identified six QTLs associated with FLA, which were located on chromosomes 1, 2, 4, 5, 6, and 9, respectively (Figure 2). Based on the Gramene website (http://www.gramene.org/markers/), the BLAST (http://blast.ncbi.nlm.nih.gov/Blast.cgi), and the China Rice Data Center database (http://www.ricedata.cn/gene/list/1499.htm), we compared the QTLs identified in this study with the QTLs reported previously controlling FLA. The position range of five QTLs was overlapped with the flanking regions of four QTLs and one gene (*OsLIC*) reported previously (Li et al., 1999; Dong et al., 2003; Wang et al., 2008; Zhang et al., 2012, 2013; Ham et al., 2019) (Supplementary Table 12), and the remaining one QTL *qFLA2* was newly identified in this study.

Two GWAS signals significantly associated with FLA were identified to nearly single-gene resolution. Gene *OsFLA6* coincided with the locations of the gene, *OsLIC*. Wang et al. (2008) reported that inhibition of endogenous *OsLIC* expression resulted in a significant increase in leaf angle and tiller angle. Zhang et al. (2012) confirmed that *OsLIC* modulated the leaf angle by acting as an antagonistic transcription factor of *BRASSINAZOLE-RESISTANT 1* (*BZR1*) *via* the brassinosteroid signaling pathway. In this study, we further confirmed that *OsFLA6* (*OsLIC*) could regulate the FLA. Gene *OsFLA2* is a newly identified gene in this study. The full length of *OsFLA2* is 2,177 bp, including three exons and two introns. Gene *OsFLA2* encodes a 95 amino acid protein. We have demonstrated that a base C-to-T nonsynonymous mutation at nt 137 in the cDNA sequence of *OsFLA2* caused the large FLA phenotype by qRT-PCR, complementation test, and hybrid F_1_ seed production in the field.

For *OsFLA2*, the CC allele and TT allele were both present in wild rice (Figure 6 and Supplementary Figure 2). The percentage of the CC allele was greater than that of the TT allele. We called the CC allele an ancestral allele and the TT allele a derived allele. Except for TRJ, as the latitude increases, the percentage of the CC allele was still greater than the TT allele. For TRJ, the percentage of the TT allele was greater than that of the CC allele. For *OsFLA6*, the GG allele and AA allele were both present in wild rice (Figure 6 and Supplementary Figure 2). The percentage of the GG allele was greater than that of the AA allele. The GG allele was the ancestral allele, and the AA allele was the derived allele. Except for TRJ, as the latitude increases, the percentage of the AA allele was greater than GG allele. For TRJ, the percentage of the GG allele was greater than that of the AA allele. The allele frequency distribution in TRJ was different from that in other subgroups, and the dominant allele GG was the one that controls the large FLA. We speculated that the reason may be due to tropical japonica growth in low-latitude high-altitude mountains in the field, and the light intensity is weaker than that in the plain. The small FLA of temperate *japonica* rice is beneficial for obtaining high grain yield in pure-line cultivars (Yang et al., 1984) but is adverse for hybrid seed production due to the requirement of removing the flag leaf of male-sterile plants to receive pollen from the male parent (Dong Z. Y. et al., 2018). Therefore, it is necessary to increase the FLA to facilitate pollination in the F_1_ hybrid seed production. The accessions with the two alleles, *OsFLA2*^*TT*^ and *OsFLA6*^*GG*^, can be used to increase FLA in the maintainer lines (pollen parents used for multiplying the CMS lines) of hybrid *japonica* rice using the crossing and marker-assisted selection breeding method.

Although Wang et al. (2005) cloned the gene *OsWRKY11* using a T-DNA insertion mutant *lla* with a large leaf angle, the leaf angle of mutant lla was not significantly different from that of the wild type, and all the leaves of the plant were still uplifted, which was difficult to be used in hybrid F_1_ seed production. In this study, *OsFLA2* only controlled the FLA. In addition, the excellent allele of *OsFLA2* was obtained from conventional materials. The maintainer line with large FLA was selected by back-crossing, and then the male-sterile line with large FLA was developed by back-crossing, which can avoid the procedure of cutting leaves and help to realize mechanized hybrid F_1_ seed production.

## Data Availability Statement

The datasets presented in this study can be found in online repositories. The names of the repository/repositories and accession number(s) can be found in the article/Supplementary Material.

## Author Contributions

JJ, DH, and XD designed the experiments and managed the project. YuZ, YL, CH, LX, YiZ, and DW conducted field planting and phenotypic identification. YuZ, YL, and CH prepared RNA samples, qRT-PCR, and transformation analysis. JJ and LX performed the data analysis. XD and JJ wrote the manuscript draft. DH reviewed the manuscript. All authors reviewed the manuscript.

## Funding

This research was supported by the Natural Science Foundation of Anhui Province (2108085MC100) and National Natural Science Foundation of China (31571743 and 31601374).

## Conflict of Interest

The authors declare that the research was conducted in the absence of any commercial or financial relationships that could be construed as a potential conflict of interest.

## Publisher's Note

All claims expressed in this article are solely those of the authors and do not necessarily represent those of their affiliated organizations, or those of the publisher, the editors and the reviewers. Any product that may be evaluated in this article, or claim that may be made by its manufacturer, is not guaranteed or endorsed by the publisher.

## Supplementary Material

Supplementary Figure 1 Scatterplot of flag leaf angle (FLA) across different years (2017–2019) and locations (Nanjing and Hefei). (a) Phenotype correlation between 2017 Nanjing and 2018 Nanjing. (b) Phenotype correlation between 2017 Nanjing and 2019 Nanjing. (c) Phenotype correlation between 2018 Nanjing and 2019 Nanjing. (d) Phenotype correlation between 2017 Hefei and 2018 Hefei. (e) Phenotype correlation between 2017 Hefei and 2019 Hefei. (f) Phenotype correlation between 2018 Hefei and 2019 Hefei. (g) Phenotype correlation between 2017 Nanjing and 2017 Hefei. (h) Phenotype correlation between 2018 Nanjing and 2018 Hefei. (i) Phenotype correlation between 2019 Nanjing and 2019 Hefei.

Supplementary Figure 2 The allele frequency at the causal polymorphisms of *OsFLA2* and *OsFLA6*. The sequence information of 30 wild rice and 202 *Oryza sativa* was downloaded from the website of https://www.ebi.ac.uk/ena/browser/view/PRJEB2829 and http://ricevarmap.ncpgr.cn/two_cultivars_compare/, respectively.

Supplementary Table 1 A list of quantitative trait loci (QTLs) controlling flag leaf angle (FLA) published so far.

Supplementary Table 2 The 353 rice accession information used in this study and the mean phenotype value of FLA in six environments.

Supplementary Table 3 The results of joint analysis of variance for FLA.

Supplementary Table 4 The single nucleotide polymorphism (SNP) information in the 29.63–29.83 Mb candidate region for FLA.

Supplementary Table 5 Candidate gene annotation in the region 29.63–29.83 Mb associated with FLA.

Supplementary Table 6 The SNP information in the 2.27–2.47 Mb candidate region for FLA.

Supplementary Table 7 Candidate gene annotation in the region 2.27–2.47 Mb associated with FLA.

Supplementary Table 8 Base information of 30 *Oryza rufipogon* reported by Huang et al. (2012) (https://www.ebi.ac.uk/ena/browser/view/PRJEB2829).

Supplementary Table 9 Base information of 202 *Oryza sativa* downloaded from RiceVarMap version 2.0.

Supplementary Table 10 Single nucleotide polymorphism distribution of *OsFLA6* and *OsFLA2* in 30 *O. rufipogon*.

Supplementary Table 11 Single nucleotide polymorphism distribution of *OsFLA6* and *OsFLA2* in 202 *O. sativa*.

Supplementary Table 12 The results of QTLs for FLA detected in this study overlapped with the QTLs/genes of rice FLA reported previously.

## Abbreviations

CV: coefficients of variation
FLA: flag leaf angle
GBA: gene-based association
GWAS: genome-wide association study
LD: linkage disequilibrium
MAF: minimum allele frequency
MLM: mixed linear model
qRT-PCR: quantitative real-time polymerase chain reaction
QTL: quantitative trait loci
SNP: single nucleotide polymorphism.
