# Supplementary figures and images for: An Analysis of Natural Variation Reveals That OsFLA2 Controls Flag Leaf Angle in Rice (Oryza sativa L.)
Source: Front Plant Sci. 2022 Jun 23;13:906912. doi: 10.3389/fpls.2022.906912 (PMC9260283; doi:10.3389/fpls.2022.906912)

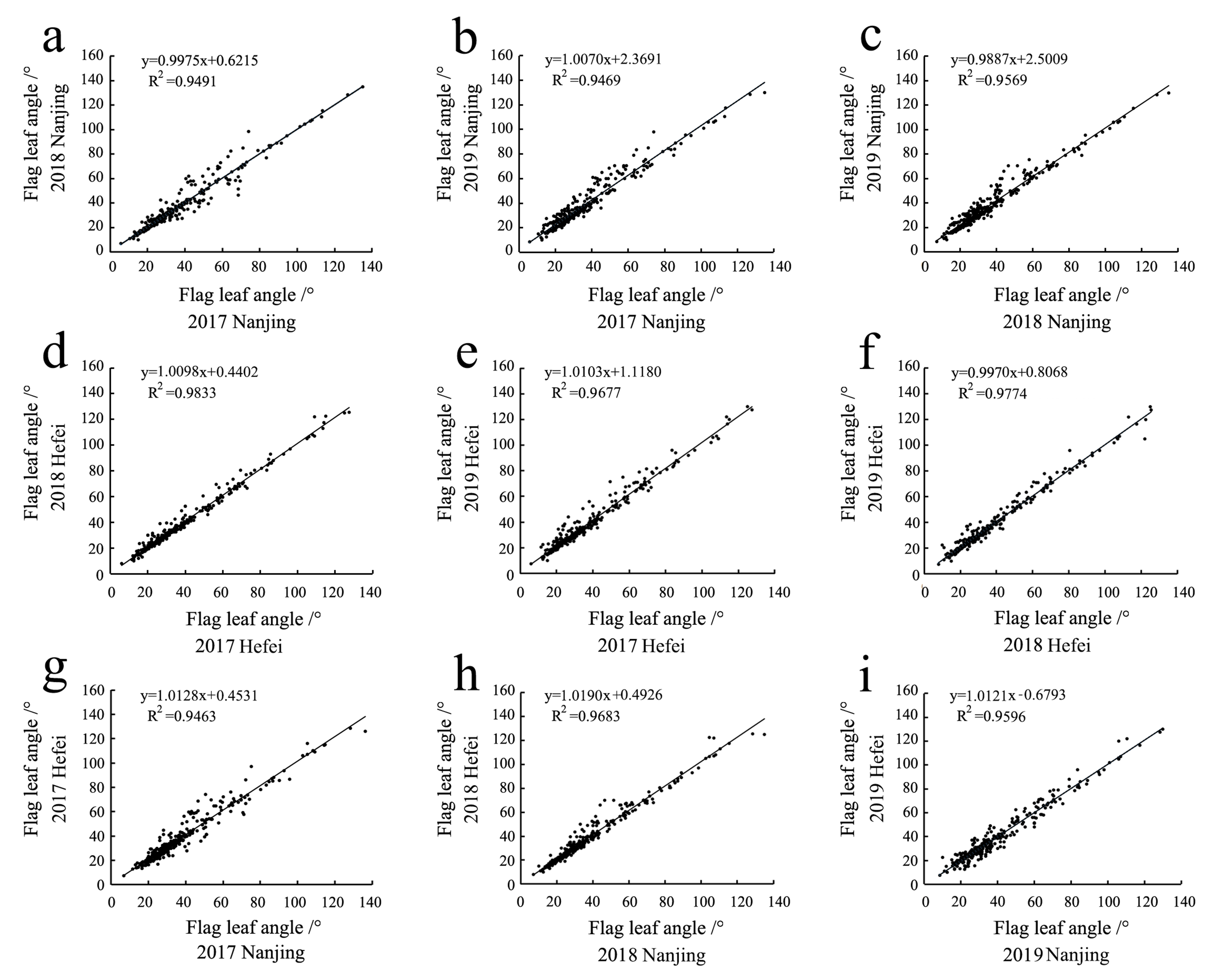

Supplement: Supplementary Figure 1 — Scatterplot of flag leaf angle (FLA) across different years (2017–2019) and locations (Nanjing and Hefei). (a) Phenotype correlation between 2017 Nanjing and 2018 Nanjing. (b) Phenotype correlation between 2017 Nanjing and 2019 Nanjing. (c) Phenotype correlation between 2018 Nanjing and 2019 Nanjing. (d) Phenotype correlation between 2017 Hefei and 2018 Hefei. (e) Phenotype correlation between 2017 Hefei and 2019 Hefei. (f) Phenotype correlation between 2018 Hefei and 2019 Hefei. (g) Phenotype correlation between 2017 Nanjing and 2017 Hefei. (h) Phenotype correlation between 2018 Nanjing and 2018 Hefei. (i) Phenotype correlation between 2019 Nanjing and 2019 Hefei. [file Image_1.TIF]

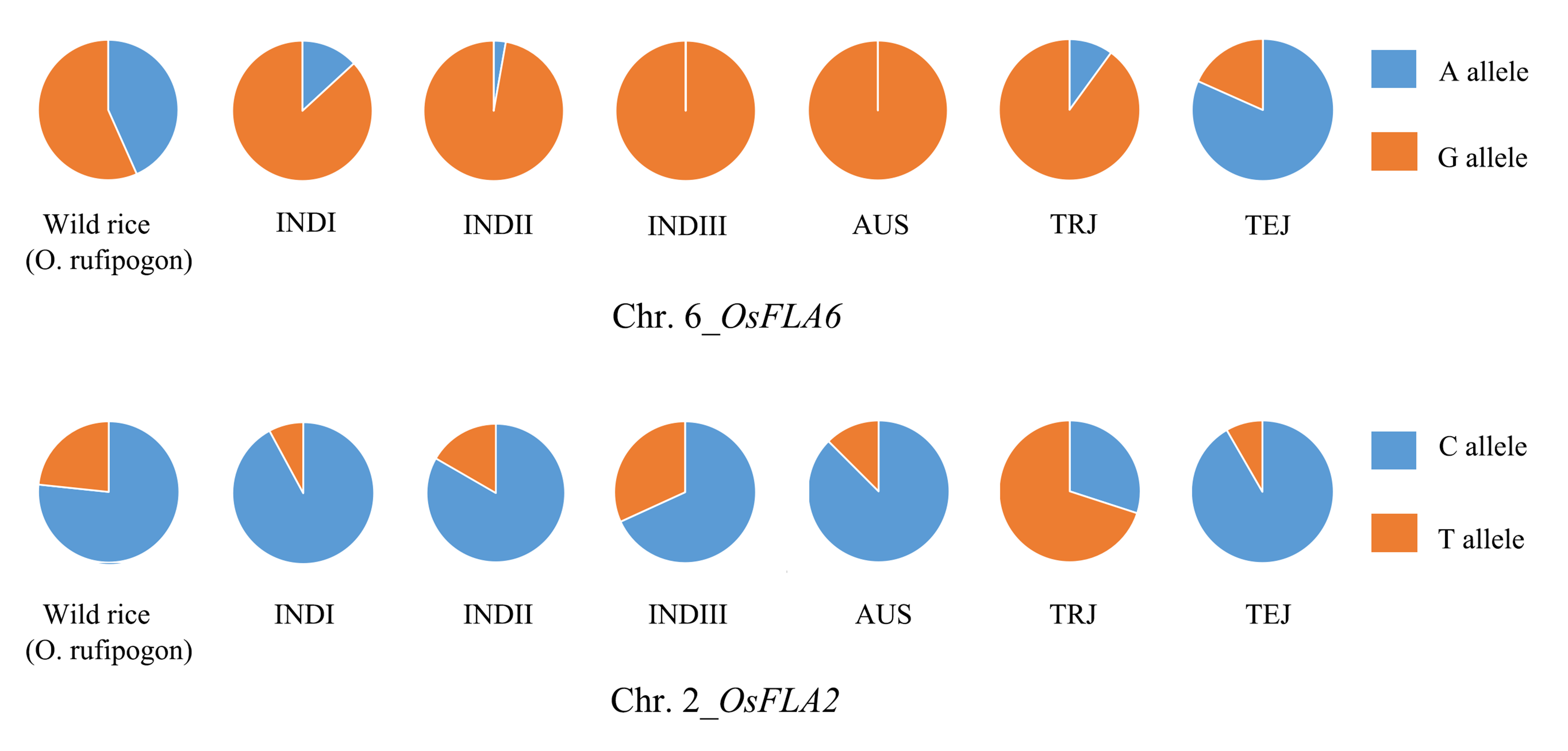

Supplement: Supplementary Figure 2 — The allele frequency at the causal polymorphisms of OsFLA2 and OsFLA6. The sequence information of 30 wild rice and 202 Oryza sativa was downloaded from the website of https://www.ebi.ac.uk/ena/browser/view/PRJEB2829 and http://ricevarmap.ncpgr.cn/two_cultivars_compare/, respectively. [file Image_2.TIF]
