# Supplementary material for: An Analysis of Natural Variation Reveals That OsFLA2 Controls Flag Leaf Angle in Rice (Oryza sativa L.)
Source: Front Plant Sci. 2022 Jun 23;13:906912. doi: 10.3389/fpls.2022.906912 (PMC9260283; doi:10.3389/fpls.2022.906912)
Supplement: Supplementary Table 1 — A list of quantitative trait loci (QTLs) controlling flag leaf angle (FLA) published so far. [file Table_1.DOC]

**Table S1.** A list of QTLs controlling flag leaf angle published so far.

| Trait | Chromosome | | | | | | | | | | | | Reference | QTLs analytical  method |
| --- | --- | --- | --- | --- | --- | --- | --- | --- | --- | --- | --- | --- | --- | --- |
|  | 1 | 2 | 3 | 4 | 5 | 6 | 7 | 8 | 9 | 10 | 11 | 12 |  |  |
| FLA |  | 1 |  |  | 1 | 1 | 1 |  | 2 |  |  |  | Li et al. 1999 | Linkage analysis |
|  | 1 |  |  | 1 |  | 1 |  |  | 1 |  | 2 |  | Yan et al. 1999 | Linkage analysis |
|  | 1 | 1 | 1 |  |  |  |  |  |  |  |  | 1 | Dong et al. 2003 | Linkage analysis |
|  | 2 | 1 |  | 1 |  |  |  | 1 |  |  |  |  | Kobayashi et al. 2003 | Linkage analysis |
|  | 1 |  | 1 |  |  | 2 |  | 1 |  |  |  |  | Luo et al. 2008 | Linkage analysis |
|  | 2 |  | 1 |  | 1 |  |  |  |  |  | 1 |  | Zhang et al. 2008 | Linkage analysis |
|  | 1 | 2 |  |  |  | 1 | 1 | 1 | 1 |  |  | 2 | Cai 2009 | Linkage analysis |
|  |  | 1 | 2 |  |  |  |  | 1 |  |  |  |  | Hu et al. 2012 | Linkage analysis |
|  | 1 | 1 | 1 |  |  |  |  |  | 1 |  |  |  | Wang et al. 2012 | Linkage analysis |
|  |  |  |  | 1 | 1 |  |  |  | 1 |  | 1 |  | Zhang et al. 2013 | Linkage analysis |
|  |  |  |  |  | 1 |  |  | 1 |  |  |  |  | Bian et al. 2014 | Linkage analysis |
|  |  |  |  |  |  |  | 2 |  |  |  |  |  | Zou et al. 2014 | Linkage analysis |
|  |  |  |  |  |  |  |  | 2 |  |  |  |  | Zhu et al. 2016 | Linkage analysis |
|  |  |  |  | 1 |  |  |  |  |  |  | 4 |  | Ham et al. 2019 | Linkage analysis |
|  |  |  |  |  |  |  |  |  |  |  |  |  |  |  |
|  | 3 |  |  | 1 | 1 |  |  |  |  |  |  |  | Huang et al. 2010 | Association analysis |
|  |  | 5 |  |  | 1 |  |  |  | 1 | 1 |  |  | Chen et al. 2012 | Association analysis |
|  | 2 | 2 | 1 | 3 | 1 |  | 1 |  | 1 |  | 1 |  | Lu et al. 2015 | Association analysis |
|  | 8 | 6 | 5 | 5 | 3 | 6 | 5 | 10 | 4 | 3 | 1 | 4 | Dong et al. 2018a | Association analysis |
|  | 2 |  | 4 | 3 |  |  | 1 | 1 |  | 2 | 1 | 1 | Dong et al. 2018b | Association analysis |
| Total | 24 | 20 | 16 | 16 | 10 | 11 | 11 | 18 | 12 | 6 | 11 | 8 |  |  |
