# Supplementary material for: An Analysis of Natural Variation Reveals That OsFLA2 Controls Flag Leaf Angle in Rice (Oryza sativa L.)
Source: Front Plant Sci. 2022 Jun 23;13:906912. doi: 10.3389/fpls.2022.906912 (PMC9260283; doi:10.3389/fpls.2022.906912)
Supplement: Supplementary Table 3 — The results of joint analysis of variance for FLA. [file Table_3.DOC]

**Table S3.** The results of joint analysis of variance for the FLA traits.

| Traits | Source of variation | df | SS | MS | *F*-value | *F*_0.05_ | *F*_0.01_ |
| --- | --- | --- | --- | --- | --- | --- | --- |
| FLA/° | Genotypes | 352 | 1910966.93 | 5428.88 | 166.43** | 1.18 | 1.26 |
|  | Environments | 5 | 3916.18 | 783.24 | 3.74 | 4.39 | 8.75 |
|  | Genotype × Environment | 1760 | 57409.49 | 32.62 | 1.40** | 1.12 | 1.17 |

df, degrees of freedom; SS, sum of squares; MS, mean square.** Significant differences at *P* < 0.01. FLA, flag leaf angle.
