# Supplementary material for: An Analysis of Natural Variation Reveals That OsFLA2 Controls Flag Leaf Angle in Rice (Oryza sativa L.)
Source: Front Plant Sci. 2022 Jun 23;13:906912. doi: 10.3389/fpls.2022.906912 (PMC9260283; doi:10.3389/fpls.2022.906912)
Supplement: Supplementary Table 4 — The single nucleotide polymorphism (SNP) information in the 29.63–29.83 Mb candidate region for FLA. [file Table_4.DOC]

**Table S4.** The SNP information in the 29.63-29.83 Mb candidate region for flag leaf angle.

| Gene ID | MSU ID | SNP location | Reference | Alterative | Region | Variation type | Associated signal in GWAS (P-value) | Gene-based association with traits |
| --- | --- | --- | --- | --- | --- | --- | --- | --- |
|
| Os06g0702500 | LOC_Os06g48940 | 6:29630487 | A | G | intron |  |  |  |
| Os06g0702500 | LOC_Os06g48940 | 6:29631025 | C | T | exon | synonymous |  |  |
| Os06g0702500 | LOC_Os06g48940 | 6:29631565 | C | A | iIntron |  |  |  |
| Os06g0702500 | LOC_Os06g48940 | 6:29631724 | G | A | intron |  |  |  |
| Os06g0702500 | LOC_Os06g48940 | 6:29631821 | A | G | exon | synonymous |  |  |
| Os06g0702500 | LOC_Os06g48940 | 6:29634260 | G | A | 3_prime_UTR |  |  |  |
| Os06g0702500 | LOC_Os06g48940 | 6:29634548 | C | T | 3_prime_UTR |  |  |  |
| Os06g0702500 | LOC_Os06g48940 | 6:29634549 | C | G | 3_prime_UTR |  |  |  |
| Os06g0702500 | LOC_Os06g48940 | 6:29634566 | C | T | 3_prime_UTR |  |  |  |
| Os06g0702500 | LOC_Os06g48940 | 6:29634644 | T | C | 3_prime_UTR |  |  |  |
| Os06g0702500 | LOC_Os06g48940 | 6:29634661 | C | T | 3_prime_UTR |  |  |  |
| Os06g0702500 | LOC_Os06g48940 | 6:29634719 | C | T | downstream |  |  |  |
| Os06g0702500 | LOC_Os06g48940 | 6:29634793 | A | G | downstream |  |  |  |
| Os06g0702500 | LOC_Os06g48940 | 6:29634842 | G | A | downstream |  |  |  |
| Os06g0702500 | LOC_Os06g48940 | 6:29634887 | G | A | downstream |  |  |  |
| Os06g0702500 | LOC_Os06g48940 | 6:29634966 | G | A | downstream |  |  |  |
| Os06g0702500 | LOC_Os06g48940 | 6:29635558 | A | G | downstream |  |  |  |
| Os06g0702500 | LOC_Os06g48940 | 6:29635619 | C | T | downstream |  |  |  |
| Os06g0702500 | LOC_Os06g48940 | 6:29635625 | A | G | downstream |  |  |  |
| Os06g0702500 | LOC_Os06g48940 | 6:29635741 | C | A | downstream |  |  |  |
| Os06g0702500 | LOC_Os06g48940 | 6:29635928 | C | T | downstream |  |  |  |
| Os06g0702500 | LOC_Os06g48940 | 6:29635939 | C | T | downstream |  |  |  |
| Os06g0702500 | LOC_Os06g48940 | 6:29635959 | A | G | downstream |  |  |  |
| Os06g0702500 | LOC_Os06g48940 | 6:29636092 | G | A | downstream |  |  |  |
| Os06g0702500 | LOC_Os06g48940 | 6:29636238 | A | C | downstream |  |  |  |
| Os06g0702500 | LOC_Os06g48940 | 6:29636414 | G | A | downstream |  |  |  |
| Os06g0702500 | LOC_Os06g48940 | 6:29636415 | C | T | downstream |  |  |  |
| Os06g0702500 | LOC_Os06g48940 | 6:29636442 | G | A | downstream |  |  |  |
| Os06g0702500 | LOC_Os06g48940 | 6:29636495 | T | G | downstream |  |  |  |
| Os06g0702500 | LOC_Os06g48940 | 6:29636516 | T | A | downstream |  |  |  |
| Os06g0702500 | LOC_Os06g48940 | 6:29636751 | G | A | downstream |  |  |  |
| Os06g0702500 | LOC_Os06g48940 | 6:29636766 | C | T | downstream |  |  |  |
| Os06g0702500 | LOC_Os06g48940 | 6:29636904 | C | T | downstream |  |  |  |
| Os06g0702500 | LOC_Os06g48940 | 6:29636965 | T | C | downstream |  |  |  |
| Os06g0702500 | LOC_Os06g48940 | 6:29638127 | G | A | downstream |  |  |  |
| Os06g0702500 | LOC_Os06g48940 | 6:29638166 | G | A | downstream |  |  |  |
| Os06g0702500 | LOC_Os06g48940 | 6:29638211 | A | G | downstream |  |  |  |
| Os06g0702500 | LOC_Os06g48940 | 6:29638251 | T | C | downstream |  |  |  |
| Os06g0702500 | LOC_Os06g48940 | 6:29638542 | A | T | downstream |  |  |  |
| Os06g0702500 | LOC_Os06g48940 | 6:29638602 | A | G | downstream |  |  |  |
| Os06g0702500 | LOC_Os06g48940 | 6:29638798 | C | A | downstream |  |  |  |
| Os06g0702500 | LOC_Os06g48940 | 6:29638803 | A | G | downstream |  |  |  |
| Os06g0702500 | LOC_Os06g48940 | 6:29638912 | C | A | downstream |  |  |  |
| Os06g0702500 | LOC_Os06g48940 | 6:29638924 | G | T | downstream |  |  |  |
| Os06g0702500 | LOC_Os06g48940 | 6:29639042 | A | G | downstream |  |  |  |
| Os06g0702500 | LOC_Os06g48940 | 6:29639240 | T | C | downstream |  |  |  |
| Os06g0702500 | LOC_Os06g48940 | 6:29639246 | T | A | downstream |  |  |  |
| Os06g0702500 | LOC_Os06g48940 | 6:29639254 | A | G | downstream |  |  |  |
| Os06g0702500 | LOC_Os06g48940 | 6:29639257 | T | C | downstream |  |  |  |
| Os06g0702500 | LOC_Os06g48940 | 6:29639319 | T | C | downstream |  |  |  |
| Os06g0702500 | LOC_Os06g48940 | 6:29639391 | G | A | downstream |  |  |  |
| Os06g0702500 | LOC_Os06g48940 | 6:29639436 | G | A | downstream |  |  |  |
| Os06g0702500 | LOC_Os06g48940 | 6:29639617 | C | T | downstream |  |  |  |
| Os06g0702500 | LOC_Os06g48940 | 6:29639630 | A | T | downstream |  |  |  |
| Os06g0702600 | LOC_Os06g48950 | 6:29652137 | G | A | upstream |  |  |  |
| Os06g0702600 | LOC_Os06g48950 | 6:29652172 | G | A | upstream |  |  |  |
| Os06g0702600 | LOC_Os06g48950 | 6:29652411 | C | G | upstream |  |  |  |
| Os06g0702600 | LOC_Os06g48950 | 6:29652772 | C | T | upstream |  |  |  |
| Os06g0702600 | LOC_Os06g48950 | 6:29652804 | C | T | upstream |  |  |  |
| Os06g0702600 | LOC_Os06g48950 | 6:29652890 | T | C | upstream |  |  |  |
| Os06g0702600 | LOC_Os06g48950 | 6:29653167 | C | T | upstream |  |  |  |
| Os06g0702600 | LOC_Os06g48950 | 6:29653182 | G | A | upstream |  |  |  |
| Os06g0702600 | LOC_Os06g48950 | 6:29653253 | C | T | upstream |  |  |  |
| Os06g0702600 | LOC_Os06g48950 | 6:29653274 | T | G | upstream |  |  |  |
| Os06g0702600 | LOC_Os06g48950 | 6:29653504 | A | C | upstream |  |  |  |
| Os06g0702600 | LOC_Os06g48950 | 6:29653896 | G | A | upstream |  |  |  |
| Os06g0702600 | LOC_Os06g48950 | 6:29653899 | A | T | upstream |  |  |  |
| Os06g0702600 | LOC_Os06g48950 | 6:29653963 | A | G | upstream |  |  |  |
| Os06g0702600 | LOC_Os06g48950 | 6:29654178 | G | A | upstream |  |  |  |
| Os06g0702600 | LOC_Os06g48950 | 6:29654220 | C | T | upstream |  |  |  |
| Os06g0702600 | LOC_Os06g48950 | 6:29654332 | C | T | upstream |  |  |  |
| Os06g0702600 | LOC_Os06g48950 | 6:29654674 | C | A | upstream |  |  |  |
| Os06g0702600 | LOC_Os06g48950 | 6:29655127 | C | G | upstream |  |  |  |
| Os06g0702600 | LOC_Os06g48950 | 6:29655458 | T | G | upstream |  |  |  |
| Os06g0702600 | LOC_Os06g48950 | 6:29655578 | C | A | upstream |  |  |  |
| Os06g0702600 | LOC_Os06g48950 | 6:29655731 | C | T | upstream |  |  |  |
| Os06g0702600 | LOC_Os06g48950 | 6:29655739 | G | T | upstream |  |  |  |
| Os06g0702600 | LOC_Os06g48950 | 6:29656324 | G | T | upstream |  |  |  |
| Os06g0702600 | LOC_Os06g48950 | 6:29656617 | C | A | upstream |  |  |  |
| Os06g0702600 | LOC_Os06g48950 | 6:29656686 | A | G | upstream |  |  |  |
| Os06g0702600 | LOC_Os06g48950 | 6:29657204 | C | T | 5_prime_UTR |  |  |  |
| Os06g0702600 | LOC_Os06g48950 | 6:29657293 | A | T | 5_prime_UTR |  |  |  |
| Os06g0702600 | LOC_Os06g48950 | 6:29657589 | T | C | intron |  |  |  |
| Os06g0702600 | LOC_Os06g48950 | 6:29657988 | C | T | intron |  |  |  |
| Os06g0702600 | LOC_Os06g48950 | 6:29658183 | T | A | intron |  |  |  |
| Os06g0702600 | LOC_Os06g48950 | 6:29658310 | C | A | intron |  |  |  |
| Os06g0702600 | LOC_Os06g48950 | 6:29658347 | A | C | intron |  |  |  |
| Os06g0702600 | LOC_Os06g48950 | 6:29659390 | T | C | intron |  |  |  |
| Os06g0702600 | LOC_Os06g48950 | 6:29659433 | G | T | intron |  |  |  |
| Os06g0702600 | LOC_Os06g48950 | 6:29659434 | A | G | intron |  |  |  |
| Os06g0702600 | LOC_Os06g48950 | 6:29660525 | C | T | exon | synonymous |  |  |
| Os06g0702600 | LOC_Os06g48950 | 6:29660846 | C | T | exon | synonymous |  |  |
| Os06g0702600 | LOC_Os06g48950 | 6:29661482 | C | T | exon | synonymous |  |  |
| Os06g0702600 | LOC_Os06g48950 | 6:29663352 | A | C | exon | nonsynonymous |  |  |
| Os06g0702600 | LOC_Os06g48950 | 6:29664365 | T | C | exon | synonymous |  |  |
| Os06g0702600 | LOC_Os06g48950 | 6:29665161 | T | C | downstream |  |  |  |
| Os06g0702700 | LOC_Os06g48960 | 6:29660628 | G | A | downstream |  |  |  |
| Os06g0702700 | LOC_Os06g48960 | 6:29661322 | G | A | downstream |  |  |  |
| Os06g0702700 | LOC_Os06g48960 | 6:29661637 | T | C | downstream |  |  |  |
| Os06g0702700 | LOC_Os06g48960 | 6:29662930 | A | T | downstream |  |  |  |
| Os06g0702700 | LOC_Os06g48960 | 6:29662999 | A | C | downstream |  |  |  |
| Os06g0702700 | LOC_Os06g48960 | 6:29663284 | C | T | downstream |  |  |  |
| Os06g0702700 | LOC_Os06g48960 | 6:29663797 | A | T | downstream |  |  |  |
| Os06g0702700 | LOC_Os06g48960 | 6:29663900 | A | T | downstream |  |  |  |
| Os06g0702700 | LOC_Os06g48960 | 6:29664506 | A | C | downstream |  |  |  |
| Os06g0702700 | LOC_Os06g48960 | 6:29667348 | A | T | 5_prime_UTR |  |  |  |
| Os06g0702700 | LOC_Os06g48960 | 6:29667405 | A | G | upstream |  |  |  |
| Os06g0702700 | LOC_Os06g48960 | 6:29667633 | C | T | upstream |  |  |  |
| Os06g0702700 | LOC_Os06g48960 | 6:29668905 | G | A | upstream |  |  |  |
| Os06g0702700 | LOC_Os06g48960 | 6:29669352 | C | G | upstream |  |  |  |
| Os06g0702700 | LOC_Os06g48960 | 6:29669390 | G | A | upstream |  |  |  |
| Os06g0702700 | LOC_Os06g48960 | 6:29669751 | T | C | upstream |  |  |  |
| Os06g0702700 | LOC_Os06g48960 | 6:29670365 | G | T | upstream |  |  |  |
| Os06g0702700 | LOC_Os06g48960 | 6:29670367 | A | C | upstream |  |  |  |
| Os06g0702700 | LOC_Os06g48960 | 6:29671063 | T | G | upstream |  |  |  |
| Os06g0702700 | LOC_Os06g48960 | 6:29671073 | C | T | upstream |  |  |  |
| Os06g0702700 | LOC_Os06g48960 | 6:29671597 | T | C | upstream |  |  |  |
| Os06g0702700 | LOC_Os06g48960 | 6:29672012 | G | T | upstream |  |  |  |
| Os06g0702800 | LOC_Os06g48970 | 6:29666339 | C | T | upstream |  |  |  |
| Os06g0702800 | LOC_Os06g48970 | 6:29666356 | A | T | upstream |  |  |  |
| Os06g0702800 | LOC_Os06g48970 | 6:29666461 | T | C | upstream |  |  |  |
| Os06g0702800 | LOC_Os06g48970 | 6:29672088 | C | T | exon | synonymous |  |  |
| Os06g0702800 | LOC_Os06g48970 | 6:29672539 | T | C | intron |  |  |  |
| Os06g0702800 | LOC_Os06g48970 | 6:29672772 | T | C | exon | synonymous |  |  |
| Os06g0702800 | LOC_Os06g48970 | 6:29674405 | G | A | downstream |  |  |  |
| Os06g0702800 | LOC_Os06g48970 | 6:29674439 | C | T | downstream |  |  |  |
| Os06g0702800 | LOC_Os06g48970 | 6:29674590 | T | C | downstream |  |  |  |
| Os06g0702800 | LOC_Os06g48970 | 6:29674592 | G | A | downstream |  |  |  |
| Os06g0702800 | LOC_Os06g48970 | 6:29674865 | G | A | downstream |  |  |  |
| Os06g0702800 | LOC_Os06g48970 | 6:29674930 | A | G | downstream |  |  |  |
| Os06g0702800 | LOC_Os06g48970 | 6:29675027 | G | T | downstream |  |  |  |
| Os06g0702800 | LOC_Os06g48970 | 6:29675104 | A | C | downstream |  |  |  |
| Os06g0702800 | LOC_Os06g48970 | 6:29675775 | T | A | downstream |  |  |  |
| Os06g0702800 | LOC_Os06g48970 | 6:29675996 | A | G | downstream |  |  |  |
| Os06g0702800 | LOC_Os06g48970 | 6:29676836 | A | G | downstream |  |  |  |
| Os06g0702800 | LOC_Os06g48970 | 6:29676845 | G | A | downstream |  |  |  |
| Os06g0702800 | LOC_Os06g48970 | 6:29676846 | C | T | downstream |  |  |  |
| Os06g0703000 | LOC_Os06g48980 | 6:29676253 | C | T | intron |  |  |  |
| Os06g0703000 | LOC_Os06g48980 | 6:29676288 | G | A | exon | synonymous |  |  |
| Os06g0703000 | LOC_Os06g48980 | 6:29676959 | T | G | exon | nonsynonymous |  |  |
| Os06g0703000 | LOC_Os06g48980 | 6:29677095 | A | T | exon | nonsynonymous |  |  |
| Os06g0703000 | LOC_Os06g48980 | 6:29677802 | G | A | exon | nonsynonymous |  |  |
| Os06g0703000 | LOC_Os06g48980 | 6:29677905 | T | C | 5_prime_UTR |  |  |  |
| Os06g0703000 | LOC_Os06g48980 | 6:29677969 | T | C | 5_prime_UTR |  |  |  |
| Os06g0703000 | LOC_Os06g48980 | 6:29678058 | C | T | 5_prime_UTR |  |  |  |
| Os06g0703000 | LOC_Os06g48980 | 6:29678229 | A | G | upstream |  |  |  |
| Os06g0703000 | LOC_Os06g48980 | 6:29678281 | A | G | upstream |  |  |  |
| Os06g0703000 | LOC_Os06g48980 | 6:29678475 | G | A | upstream |  |  |  |
| Os06g0703000 | LOC_Os06g48980 | 6:29678682 | A | C | upstream |  |  |  |
| Os06g0703000 | LOC_Os06g48980 | 6:29678694 | T | C | upstream |  |  |  |
| Os06g0703000 | LOC_Os06g48980 | 6:29678888 | C | T | upstream |  |  |  |
| Os06g0703000 | LOC_Os06g48980 | 6:29679007 | A | G | upstream |  |  |  |
| Os06g0703000 | LOC_Os06g48980 | 6:29679123 | C | A | upstream |  |  |  |
| Os06g0703000 | LOC_Os06g48980 | 6:29682855 | A | G | upstream |  |  |  |
| Os06g0703200 | LOC_Os06g48990 | 6:29679524 | A | G | 3_prime_UTR |  |  |  |
| Os06g0703200 | LOC_Os06g48990 | 6:29679563 | T | C | 3_prime_UTR |  |  |  |
| Os06g0703200 | LOC_Os06g48990 | 6:29679832 | A | C | intron |  |  |  |
| Os06g0703200 | LOC_Os06g48990 | 6:29679952 | T | G | exon | nonsynonymous |  |  |
| Os06g0703200 | LOC_Os06g48990 | 6:29679957 | G | T | exon | nonsynonymous |  |  |
| Os06g0703200 | LOC_Os06g48990 | 6:29679972 | T | C | exon | nonsynonymous |  |  |
| Os06g0703200 | LOC_Os06g48990 | 6:29680259 | A | G | exon | synonymous |  |  |
| Os06g0703200 | LOC_Os06g48990 | 6:29681886 | C | T | exon | nonsynonymous |  |  |
| Os06g0703200 | LOC_Os06g48990 | 6:29682145 | C | T | exon | nonsynonymous |  |  |
| Os06g0703200 | LOC_Os06g48990 | 6:29682424 | G | A | stop_gained |  |  |  |
| Os06g0703200 | LOC_Os06g48990 | 6:29683231 | A | G | upstream |  |  |  |
| Os06g0703200 | LOC_Os06g48990 | 6:29683288 | T | A | upstream |  |  |  |
| Os06g0703200 | LOC_Os06g48990 | 6:29683327 | G | A | upstream |  |  |  |
| Os06g0703200 | LOC_Os06g48990 | 6:29683384 | G | A | upstream |  |  |  |
| Os06g0703200 | LOC_Os06g48990 | 6:29683593 | C | T | upstream |  |  |  |
| Os06g0703200 | LOC_Os06g48990 | 6:29683981 | C | T | upstream |  |  |  |
| Os06g0703200 | LOC_Os06g48990 | 6:29684449 | G | T | upstream |  |  |  |
| Os06g0703200 | LOC_Os06g48990 | 6:29684550 | T | A | upstream |  |  |  |
| Os06g0703200 | LOC_Os06g48990 | 6:29684592 | G | A | upstream |  |  |  |
| Os06g0703200 | LOC_Os06g48990 | 6:29685384 | A | T | upstream |  |  |  |
| Os06g0703200 | LOC_Os06g48990 | 6:29685387 | A | C | upstream |  |  |  |
| Os06g0703200 | LOC_Os06g48990 | 6:29685481 | T | C | upstream |  |  |  |
| Os06g0703200 | LOC_Os06g48990 | 6:29685664 | C | T | upstream |  |  |  |
| Os06g0703200 | LOC_Os06g48990 | 6:29685685 | C | T | upstream |  |  |  |
| Os06g0703200 | LOC_Os06g48990 | 6:29685838 | C | T | upstream |  |  |  |
| Os06g0703200 | LOC_Os06g48990 | 6:29685861 | T | C | upstream |  |  |  |
| Os06g0703200 | LOC_Os06g48990 | 6:29685945 | T | C | upstream |  |  |  |
| Os06g0703200 | LOC_Os06g48990 | 6:29685977 | C | T | upstream |  |  |  |
| Os06g0703200 | LOC_Os06g48990 | 6:29686337 | T | C | upstream |  |  |  |
| Os06g0703200 | LOC_Os06g48990 | 6:29686998 | A | C | upstream |  |  |  |
| Os06g0703200 | LOC_Os06g48990 | 6:29687001 | A | G | upstream |  |  |  |
| Os06g0703200 | LOC_Os06g48990 | 6:29687011 | C | T | upstream |  |  |  |
| Os06g0703200 | LOC_Os06g48990 | 6:29687022 | C | T | upstream |  |  |  |
| Os06g0703300 | LOC_Os06g49000 | 6:29691967 | T | C | upstream |  |  |  |
| Os06g0703300 | LOC_Os06g49000 | 6:29692347 | G | A | upstream |  |  |  |
| Os06g0703300 | LOC_Os06g49000 | 6:29692724 | C | T | upstream |  |  |  |
| Os06g0703300 | LOC_Os06g49000 | 6:29692858 | A | C | upstream |  |  |  |
| Os06g0703300 | LOC_Os06g49000 | 6:29692946 | G | A | upstream |  |  |  |
| Os06g0703300 | LOC_Os06g49000 | 6:29692964 | T | C | upstream |  |  |  |
| Os06g0703300 | LOC_Os06g49000 | 6:29693132 | C | A | upstream |  |  |  |
| Os06g0703300 | LOC_Os06g49000 | 6:29693136 | A | G | upstream |  |  |  |
| Os06g0703300 | LOC_Os06g49000 | 6:29693312 | A | G | upstream |  |  |  |
| Os06g0703300 | LOC_Os06g49000 | 6:29693560 | C | T | upstream |  |  |  |
| Os06g0703300 | LOC_Os06g49000 | 6:29693751 | T | C | upstream |  |  |  |
| Os06g0703300 | LOC_Os06g49000 | 6:29693807 | A | T | upstream |  |  |  |
| Os06g0703300 | LOC_Os06g49000 | 6:29693850 | G | T | upstream |  |  |  |
| Os06g0703300 | LOC_Os06g49000 | 6:29693919 | T | A | upstream |  |  |  |
| Os06g0703300 | LOC_Os06g49000 | 6:29694221 | A | T | upstream |  |  |  |
| Os06g0703300 | LOC_Os06g49000 | 6:29694228 | A | G | upstream |  |  |  |
| Os06g0703300 | LOC_Os06g49000 | 6:29694583 | A | G | upstream |  |  |  |
| Os06g0703300 | LOC_Os06g49000 | 6:29694586 | C | A | upstream |  |  |  |
| Os06g0703300 | LOC_Os06g49000 | 6:29694650 | G | C | upstream |  |  |  |
| Os06g0703300 | LOC_Os06g49000 | 6:29694711 | G | T | upstream |  |  |  |
| Os06g0703300 | LOC_Os06g49000 | 6:29694747 | T | G | upstream |  |  |  |
| Os06g0703300 | LOC_Os06g49000 | 6:29694822 | C | T | upstream |  |  |  |
| Os06g0703300 | LOC_Os06g49000 | 6:29694840 | C | T | upstream |  |  |  |
| Os06g0703300 | LOC_Os06g49000 | 6:29695452 | G | A | upstream |  |  |  |
| Os06g0703300 | LOC_Os06g49000 | 6:29695517 | A | C | upstream |  |  |  |
| Os06g0703300 | LOC_Os06g49000 | 6:29695661 | C | T | upstream |  |  |  |
| Os06g0703300 | LOC_Os06g49000 | 6:29695758 | A | G | upstream |  |  |  |
| Os06g0703300 | LOC_Os06g49000 | 6:29695807 | G | A | upstream |  |  |  |
| Os06g0703300 | LOC_Os06g49000 | 6:29695820 | G | A | upstream |  |  |  |
| Os06g0703300 | LOC_Os06g49000 | 6:29695849 | G | A | upstream |  |  |  |
| Os06g0703300 | LOC_Os06g49000 | 6:29695902 | A | T | upstream |  |  |  |
| Os06g0703300 | LOC_Os06g49000 | 6:29695985 | A | C | upstream |  |  |  |
| Os06g0703300 | LOC_Os06g49000 | 6:29696188 | A | G | upstream |  |  |  |
| Os06g0703300 | LOC_Os06g49000 | 6:29696253 | A | G | upstream |  |  |  |
| Os06g0703300 | LOC_Os06g49000 | 6:29696286 | G | A | upstream |  |  |  |
| Os06g0703300 | LOC_Os06g49000 | 6:29696561 | A | G | 5_prime_UTR |  |  |  |
| Os06g0703300 | LOC_Os06g49000 | 6:29696611 | C | G | 5_prime_UTR |  |  |  |
| Os06g0703300 | LOC_Os06g49000 | 6:29696741 | G | C | exon | nonsynonymous |  |  |
| Os06g0703300 | LOC_Os06g49000 | 6:29701157 | C | T | exon | nonsynonymous |  |  |
| Os06g0703300 | LOC_Os06g49000 | 6:29701561 | C | T | exon | synonymous |  |  |
| Os06g0703300 | LOC_Os06g49000 | 6:29701606 | C | T | exon | synonymous |  |  |
| Os06g0703300 | LOC_Os06g49000 | 6:29701964 | A | G | exon | nonsynonymous |  |  |
| Os06g0703300 | LOC_Os06g49000 | 6:29701966 | G | T | exon | nonsynonymous |  |  |
| Os06g0703300 | LOC_Os06g49000 | 6:29701970 | C | G | exon | nonsynonymous |  |  |
| Os06g0703300 | LOC_Os06g49000 | 6:29702300 | C | T | 3_prime_UTR |  |  |  |
| Os06g0703300 | LOC_Os06g49000 | 6:29702325 | T | C | 3_prime_UTR |  |  |  |
| Os06g0703300 | LOC_Os06g49000 | 6:29702712 | G | A | exon | nonsynonymous |  |  |
| Os06g0703300 | LOC_Os06g49000 | 6:29703030 | A | G | 3_prime_UTR |  |  |  |
| Os06g0703300 | LOC_Os06g49000 | 6:29703077 | C | T | 3_prime_UTR |  |  |  |
| Os06g0703300 | LOC_Os06g49000 | 6:29703129 | T | C | 3_prime_UTR |  |  |  |
| Os06g0703400 | None | 6:29697584 | A | C | downstream |  |  |  |
| Os06g0703400 | None | 6:29697909 | G | C | downstream |  |  |  |
| Os06g0703400 | None | 6:29697974 | A | G | downstream |  |  |  |
| Os06g0703400 | None | 6:29698010 | G | A | downstream |  |  |  |
| Os06g0703400 | None | 6:29698123 | C | A | downstream |  |  |  |
| Os06g0703400 | None | 6:29698288 | A | G | downstream |  |  |  |
| Os06g0703400 | None | 6:29698299 | T | A | downstream |  |  |  |
| Os06g0703400 | None | 6:29698342 | A | G | downstream |  |  |  |
| Os06g0703400 | None | 6:29698358 | A | G | downstream |  |  |  |
| Os06g0703400 | None | 6:29698514 | A | G | downstream |  |  |  |
| Os06g0703400 | None | 6:29698692 | T | C | downstream |  |  |  |
| Os06g0703400 | None | 6:29698699 | G | A | downstream |  |  |  |
| Os06g0703400 | None | 6:29698776 | T | C | downstream |  |  |  |
| Os06g0703400 | None | 6:29698788 | G | A | downstream |  |  |  |
| Os06g0703400 | None | 6:29698799 | C | T | downstream |  |  |  |
| Os06g0703400 | None | 6:29702208 | A | G | 5_prime_UTR |  |  |  |
| Os06g0703400 | None | 6:29704267 | T | C | upstream |  |  |  |
| Os06g0703400 | None | 6:29704713 | G | A | upstream |  |  |  |
| Os06g0703400 | None | 6:29704766 | A | G | upstream |  |  |  |
| Os06g0703400 | None | 6:29704953 | G | A | upstream |  |  |  |
| Os06g0703400 | None | 6:29705029 | A | G | upstream |  |  |  |
| Os06g0703400 | None | 6:29705045 | A | G | upstream |  |  |  |
| Os06g0703400 | None | 6:29705182 | G | A | upstream |  |  |  |
| Os06g0703400 | None | 6:29705842 | C | T | upstream |  |  |  |
| Os06g0703400 | None | 6:29706194 | T | A | upstream |  |  |  |
| Os06g0703400 | None | 6:29706215 | G | A | upstream |  |  |  |
| Os06g0703400 | None | 6:29707191 | C | G | upstream |  |  |  |
| Os06g0703500 | LOC_Os06g49010 | 6:29703598 | A | C | 3_prime_UTR |  |  |  |
| Os06g0703500 | LOC_Os06g49010 | 6:29703870 | A | G | exon | synonymous |  |  |
| Os06g0703500 | LOC_Os06g49010 | 6:29705372 | G | T | exon | nonsynonymous |  |  |
| Os06g0703500 | LOC_Os06g49010 | 6:29706000 | G | A | intron |  |  |  |
| Os06g0703500 | LOC_Os06g49010 | 6:29706892 | C | T | exon | nonsynonymous |  |  |
| Os06g0703500 | LOC_Os06g49010 | 6:29706995 | C | T | exon | synonymous |  |  |
| Os06g0703500 | LOC_Os06g49010 | 6:29707537 | G | T | 5_prime_UTR |  |  |  |
| Os06g0703500 | LOC_Os06g49010 | 6:29707723 | C | T | 5_prime_UTR |  |  |  |
| Os06g0703500 | LOC_Os06g49010 | 6:29708892 | G | T | upstream |  |  |  |
| Os06g0703500 | LOC_Os06g49010 | 6:29709337 | A | C | upstream |  |  |  |
| Os06g0703500 | LOC_Os06g49010 | 6:29709340 | T | C | upstream |  |  |  |
| Os06g0703500 | LOC_Os06g49010 | 6:29709368 | C | T | upstream |  |  |  |
| Os06g0703500 | LOC_Os06g49010 | 6:29709515 | A | C | upstream |  |  |  |
| Os06g0703500 | LOC_Os06g49010 | 6:29709671 | C | T | upstream |  |  |  |
| Os06g0703500 | LOC_Os06g49010 | 6:29709674 | T | C | upstream |  |  |  |
| Os06g0703500 | LOC_Os06g49010 | 6:29709750 | A | G | upstream |  |  |  |
| Os06g0703500 | LOC_Os06g49010 | 6:29709769 | A | T | upstream |  |  |  |
| Os06g0703500 | LOC_Os06g49010 | 6:29709834 | G | T | upstream |  |  |  |
| Os06g0703500 | LOC_Os06g49010 | 6:29709960 | A | G | upstream |  |  |  |
| Os06g0703500 | LOC_Os06g49010 | 6:29710191 | G | A | upstream |  |  |  |
| Os06g0703500 | LOC_Os06g49010 | 6:29710535 | A | T | upstream |  |  |  |
| Os06g0703500 | LOC_Os06g49010 | 6:29710547 | A | T | upstream |  |  |  |
| Os06g0703500 | LOC_Os06g49010 | 6:29710650 | G | T | upstream |  |  |  |
| Os06g0703500 | LOC_Os06g49010 | 6:29710784 | C | A | upstream |  |  |  |
| Os06g0703500 | LOC_Os06g49010 | 6:29710797 | T | C | upstream |  |  |  |
| Os06g0703500 | LOC_Os06g49010 | 6:29710874 | C | T | upstream |  |  |  |
| Os06g0703500 | LOC_Os06g49010 | 6:29711151 | T | C | upstream |  |  |  |
| Os06g0703500 | LOC_Os06g49010 | 6:29712748 | T | C | upstream |  |  |  |
| Os06g0703500 | LOC_Os06g49010 | 6:29712770 | G | A | upstream |  |  |  |
| Os06g0703500 | LOC_Os06g49010 | 6:29712938 | T | C | upstream |  |  |  |
| Os06g0703500 | LOC_Os06g49010 | 6:29712943 | C | A | upstream |  |  |  |
| Os06g0703500 | LOC_Os06g49010 | 6:29713379 | C | G | upstream |  |  |  |
| Os06g0703550 | None | 6:29698858 | A | G | upstream |  |  |  |
| Os06g0703550 | None | 6:29698959 | A | G | upstream |  |  |  |
| Os06g0703550 | None | 6:29699071 | G | A | upstream |  |  |  |
| Os06g0703550 | None | 6:29699089 | G | A | upstream |  |  |  |
| Os06g0703550 | None | 6:29699912 | A | G | upstream |  |  |  |
| Os06g0703550 | None | 6:29700367 | G | A | upstream |  |  |  |
| Os06g0703550 | None | 6:29700395 | A | C | upstream |  |  |  |
| Os06g0703550 | None | 6:29700512 | C | T | upstream |  |  |  |
| Os06g0703550 | None | 6:29700597 | T | A | upstream |  |  |  |
| Os06g0703550 | None | 6:29700642 | C | T | upstream |  |  |  |
| Os06g0703550 | None | 6:29700647 | G | A | upstream |  |  |  |
| Os06g0703550 | None | 6:29700692 | T | A | upstream |  |  |  |
| Os06g0703600 | LOC_Os06g49020 | 6:29711475 | T | C | 3_prime_UTR |  |  |  |
| Os06g0703600 | LOC_Os06g49020 | 6:29712607 | G | A | 5_prime_UTR |  |  |  |
| Os06g0703600 | LOC_Os06g49020 | 6:29713728 | G | A | upstream |  |  |  |
| Os06g0703600 | LOC_Os06g49020 | 6:29713890 | C | G | upstream |  |  |  |
| Os06g0703600 | LOC_Os06g49020 | 6:29714028 | C | G | upstream |  |  |  |
| Os06g0703600 | LOC_Os06g49020 | 6:29714831 | C | A | upstream |  |  |  |
| Os06g0703600 | LOC_Os06g49020 | 6:29714832 | T | C | upstream |  |  |  |
| Os06g0703600 | LOC_Os06g49020 | 6:29714941 | C | T | upstream |  |  |  |
| Os06g0703600 | LOC_Os06g49020 | 6:29715016 | A | T | upstream |  |  |  |
| Os06g0703600 | LOC_Os06g49020 | 6:29715119 | T | C | upstream |  |  |  |
| Os06g0703600 | LOC_Os06g49020 | 6:29715374 | A | C | upstream |  |  |  |
| Os06g0703600 | LOC_Os06g49020 | 6:29715478 | G | A | upstream |  |  |  |
| Os06g0703600 | LOC_Os06g49020 | 6:29715479 | C | T | upstream |  |  |  |
| Os06g0703600 | LOC_Os06g49020 | 6:29715543 | G | T | upstream |  |  |  |
| Os06g0703600 | LOC_Os06g49020 | 6:29715700 | G | A | upstream |  |  |  |
| Os06g0703600 | LOC_Os06g49020 | 6:29716003 | C | A | upstream |  |  |  |
| Os06g0703600 | LOC_Os06g49020 | 6:29717412 | C | T | upstream |  |  |  |
| Os06g0703600 | LOC_Os06g49020 | 6:29717559 | T | C | upstream |  |  |  |
| Os06g0703700 | None | 6:29707312 | C | T | upstream |  |  |  |
| Os06g0703700 | None | 6:29708376 | A | G | upstream |  |  |  |
| Os06g0703700 | None | 6:29708393 | A | C | upstream |  |  |  |
| Os06g0703700 | None | 6:29712496 | T | A | exon | nonsynonymous |  |  |
| Os06g0703700 | None | 6:29712712 | C | T | 3_prime_UTR |  |  |  |
| Os06g0703700 | None | 6:29717692 | T | A | downstream |  |  |  |
| Os06g0703800 | LOC_Os06g49030 | 6:29713605 | T | C | exon | synonymous |  |  |
| Os06g0703800 | LOC_Os06g49030 | 6:29716915 | A | G | 3_prime_UTR |  |  |  |
| Os06g0703800 | LOC_Os06g49030 | 6:29717784 | G | A | downstream |  |  |  |
| Os06g0703800 | LOC_Os06g49030 | 6:29717944 | T | C | downstream |  |  |  |
| Os06g0703800 | LOC_Os06g49030 | 6:29718016 | C | T | downstream |  |  |  |
| Os06g0703900 | LOC_Os06g49040 | 6:29718660 | T | A | upstream |  |  |  |
| Os06g0703900 | LOC_Os06g49040 | 6:29718671 | C | T | upstream |  |  |  |
| Os06g0703900 | LOC_Os06g49040 | 6:29718716 | T | C | upstream |  |  |  |
| Os06g0703900 | LOC_Os06g49040 | 6:29718744 | T | G | upstream |  |  |  |
| Os06g0703900 | LOC_Os06g49040 | 6:29718774 | C | T | upstream |  |  |  |
| Os06g0703900 | LOC_Os06g49040 | 6:29718814 | A | G | upstream |  |  |  |
| Os06g0703900 | LOC_Os06g49040 | 6:29718969 | G | A | upstream |  |  |  |
| Os06g0703900 | LOC_Os06g49040 | 6:29718994 | A | G | upstream |  |  |  |
| Os06g0703900 | LOC_Os06g49040 | 6:29719093 | T | C | upstream |  |  |  |
| Os06g0703900 | LOC_Os06g49040 | 6:29719216 | A | T | upstream |  |  |  |
| Os06g0703900 | LOC_Os06g49040 | 6:29719364 | T | G | upstream |  |  |  |
| Os06g0703900 | LOC_Os06g49040 | 6:29719551 | T | A | upstream |  |  |  |
| Os06g0703900 | LOC_Os06g49040 | 6:29719792 | G | T | upstream |  |  |  |
| Os06g0703900 | LOC_Os06g49040 | 6:29720140 | G | A | upstream |  |  |  |
| Os06g0703900 | LOC_Os06g49040 | 6:29720198 | A | G | upstream |  |  |  |
| Os06g0703900 | LOC_Os06g49040 | 6:29720250 | G | A | upstream |  |  |  |
| Os06g0703900 | LOC_Os06g49040 | 6:29720280 | T | A | upstream |  |  |  |
| Os06g0703900 | LOC_Os06g49040 | 6:29720526 | T | C | upstream |  |  |  |
| Os06g0703900 | LOC_Os06g49040 | 6:29720809 | G | A | upstream |  |  |  |
| Os06g0703900 | LOC_Os06g49040 | 6:29720936 | C | T | upstream |  |  |  |
| Os06g0703900 | LOC_Os06g49040 | 6:29720991 | G | A | upstream |  |  |  |
| Os06g0703900 | LOC_Os06g49040 | 6:29720995 | G | A | upstream |  |  |  |
| Os06g0703900 | LOC_Os06g49040 | 6:29721012 | G | T | upstream |  |  |  |
| Os06g0703900 | LOC_Os06g49040 | 6:29721040 | C | G | upstream |  |  |  |
| Os06g0703900 | LOC_Os06g49040 | 6:29721134 | C | T | upstream |  |  |  |
| Os06g0703900 | LOC_Os06g49040 | 6:29721182 | T | C | upstream |  |  |  |
| Os06g0703900 | LOC_Os06g49040 | 6:29721390 | T | G | upstream |  |  |  |
| Os06g0703900 | LOC_Os06g49040 | 6:29721533 | T | A | upstream |  |  |  |
| Os06g0703900 | LOC_Os06g49040 | 6:29721587 | C | T | upstream |  |  |  |
| Os06g0703900 | LOC_Os06g49040 | 6:29721620 | G | A | upstream |  |  |  |
| Os06g0703900 | LOC_Os06g49040 | 6:29721639 | A | G | upstream |  |  |  |
| Os06g0703900 | LOC_Os06g49040 | 6:29721661 | T | G | upstream |  |  |  |
| Os06g0703900 | LOC_Os06g49040 | 6:29721760 | T | C | upstream |  |  |  |
| Os06g0703900 | LOC_Os06g49040 | 6:29721811 | C | T | upstream |  |  |  |
| Os06g0703900 | LOC_Os06g49040 | 6:29722016 | G | A | upstream |  |  |  |
| Os06g0703900 | LOC_Os06g49040 | 6:29722499 | G | A | upstream |  |  |  |
| Os06g0703900 | LOC_Os06g49040 | 6:29722902 | T | A | upstream |  |  |  |
| Os06g0703900 | LOC_Os06g49040 | 6:29725098 | A | G | exon | nonsynonymous |  |  |
| Os06g0703900 | LOC_Os06g49040 | 6:29725724 | A | G | exon | synonymous |  |  |
| Os06g0703900 | LOC_Os06g49040 | 6:29726368 | A | G | exon | synonymous |  |  |
| Os06g0703900 | LOC_Os06g49040 | 6:29726959 | G | A | 3_prime_UTR |  |  |  |
| Os06g0703900 | LOC_Os06g49040 | 6:29727717 | A | T | downstream |  |  |  |
| Os06g0703900 | LOC_Os06g49040 | 6:29727907 | G | T | downstream |  |  |  |
| Os06g0703900 | LOC_Os06g49040 | 6:29727910 | C | T | downstream |  |  |  |
| Os06g0703900 | LOC_Os06g49040 | 6:29728403 | T | A | downstream |  |  |  |
| Os06g0703900 | LOC_Os06g49040 | 6:29728490 | T | G | downstream |  |  |  |
| Os06g0703900 | LOC_Os06g49040 | 6:29728661 | A | T | downstream |  |  |  |
| Os06g0704000 | LOC_Os06g49050 | 6:29724116 | C | T | upstream |  |  |  |
| Os06g0704000 | LOC_Os06g49050 | 6:29724128 | T | C | upstream |  |  |  |
| Os06g0704000 | LOC_Os06g49050 | 6:29727083 | T | A | upstream |  |  |  |
| Os06g0704000 | LOC_Os06g49050 | 6:29727210 | G | C | upstream |  |  |  |
| Os06g0704000 | LOC_Os06g49050 | 6:29727246 | T | G | upstream |  |  |  |
| Os06g0704000 | LOC_Os06g49050 | 6:29728748 | A | C | exon | nonsynonymous |  |  |
| Os06g0704000 | LOC_Os06g49050 | 6:29728783 | A | T | exon | nonsynonymous |  |  |
| Os06g0704000 | LOC_Os06g49050 | 6:29729061 | G | A | exon | synonymous |  |  |
| Os06g0704000 | LOC_Os06g49050 | 6:29729066 | A | G | exon | nonsynonymous |  |  |
| Os06g0704000 | LOC_Os06g49050 | 6:29729083 | T | G | exon | nonsynonymous |  |  |
| Os06g0704000 | LOC_Os06g49050 | 6:29729479 | G | T | exon | nonsynonymous |  |  |
| Os06g0704000 | LOC_Os06g49050 | 6:29731538 | C | G | exon | nonsynonymous |  |  |
| Os06g0704100 | LOC_Os06g49060 | 6:29728806 | C | T | upstream |  |  |  |
| Os06g0704100 | LOC_Os06g49060 | 6:29730665 | C | G | upstream |  |  |  |
| Os06g0704100 | LOC_Os06g49060 | 6:29730745 | C | T | upstream |  |  |  |
| Os06g0704100 | LOC_Os06g49060 | 6:29731030 | A | T | upstream |  |  |  |
| Os06g0704100 | LOC_Os06g49060 | 6:29731297 | G | C | upstream |  |  |  |
| Os06g0704100 | LOC_Os06g49060 | 6:29732280 | G | T | upstream |  |  |  |
| Os06g0704100 | LOC_Os06g49060 | 6:29732283 | C | G | upstream |  |  |  |
| Os06g0704100 | LOC_Os06g49060 | 6:29732299 | T | C | upstream |  |  |  |
| Os06g0704100 | LOC_Os06g49060 | 6:29732462 | G | A | upstream |  |  |  |
| Os06g0704100 | LOC_Os06g49060 | 6:29732574 | A | T | upstream |  |  |  |
| Os06g0704100 | LOC_Os06g49060 | 6:29732757 | G | A | upstream |  |  |  |
| Os06g0704100 | LOC_Os06g49060 | 6:29733354 | A | T | upstream |  |  |  |
| Os06g0704100 | LOC_Os06g49060 | 6:29733386 | T | C | upstream |  |  |  |
| Os06g0704100 | LOC_Os06g49060 | 6:29733431 | T | C | upstream |  |  |  |
| Os06g0704100 | LOC_Os06g49060 | 6:29733571 | A | G | upstream |  |  |  |
| Os06g0704100 | LOC_Os06g49060 | 6:29733685 | C | G | upstream |  |  |  |
| Os06g0704100 | LOC_Os06g49060 | 6:29733750 | C | A | upstream |  |  |  |
| Os06g0704100 | LOC_Os06g49060 | 6:29733807 | T | C | 5_prime_UTR |  |  |  |
| Os06g0704100 | LOC_Os06g49060 | 6:29733892 | A | G | 5_prime_UTR |  |  |  |
| Os06g0704100 | LOC_Os06g49060 | 6:29734206 | G | T | exon | nonsynonymous |  |  |
| Os06g0704100 | LOC_Os06g49060 | 6:29735976 | T | C | exon | synonymous |  |  |
| Os06g0704100 | LOC_Os06g49060 | 6:29736995 | C | T | 3_prime_UTR |  |  |  |
| Os06g0704200 | LOC_Os06g49070 | 6:29737352 | A | G | 3_prime_UTR |  |  |  |
| Os06g0704200 | LOC_Os06g49070 | 6:29737400 | C | T | 3_prime_UTR |  |  |  |
| Os06g0704200 | LOC_Os06g49070 | 6:29737406 | C | T | 3_prime_UTR |  |  |  |
| Os06g0704200 | LOC_Os06g49070 | 6:29738685 | G | A | upstream |  |  |  |
| Os06g0704200 | LOC_Os06g49070 | 6:29739564 | G | C | upstream |  |  |  |
| Os06g0704200 | LOC_Os06g49070 | 6:29739753 | G | A | upstream |  |  |  |
| Os06g0704200 | LOC_Os06g49070 | 6:29740496 | G | T | upstream |  |  |  |
| Os06g0704200 | LOC_Os06g49070 | 6:29740580 | G | A | upstream |  |  |  |
| Os06g0704200 | LOC_Os06g49070 | 6:29741583 | T | G | upstream |  |  |  |
| Os06g0704200 | LOC_Os06g49070 | 6:29741787 | A | G | upstream |  |  |  |
| Os06g0704200 | LOC_Os06g49070 | 6:29741804 | T | C | upstream |  |  |  |
| Os06g0704200 | LOC_Os06g49070 | 6:29742590 | G | T | upstream |  |  |  |
| Os06g0704200 | LOC_Os06g49070 | 6:29742724 | G | A | upstream |  |  |  |
| Os06g0704200 | LOC_Os06g49070 | 6:29742727 | G | A | upstream |  |  |  |
| Os06g0704200 | LOC_Os06g49070 | 6:29742987 | T | G | upstream |  |  |  |
| Os06g0704300 | LOC_Os06g49080 | 6:29734634 | G | A | upstream |  |  |  |
| Os06g0704300 | LOC_Os06g49080 | 6:29735447 | T | A | upstream |  |  |  |
| Os06g0704300 | LOC_Os06g49080 | 6:29736337 | T | C | upstream |  |  |  |
| Os06g0704300 | LOC_Os06g49080 | 6:29737770 | G | C | upstream |  |  |  |
| Os06g0704300 | LOC_Os06g49080 | 6:29739644 | A | G | exon | nonsynonymous | 5.11E-08 | 6.06E-04 |
| Os06g0704300 | LOC_Os06g49080 | 6:29739976 | A | G | exon | nonsynonymous |  |  |
| Os06g0704300 | LOC_Os06g49080 | 6:29740769 | G | A | exon | nonsynonymous |  |  |
| Os06g0704400 | LOC_Os06g49090 | 6:29743772 | C | T | upstream |  |  |  |
| Os06g0704400 | LOC_Os06g49090 | 6:29743780 | C | T | upstream |  |  |  |
| Os06g0704400 | LOC_Os06g49090 | 6:29744008 | T | C | upstream |  |  |  |
| Os06g0704400 | LOC_Os06g49090 | 6:29744075 | G | T | upstream |  |  |  |
| Os06g0704400 | LOC_Os06g49090 | 6:29744304 | T | A | upstream |  |  |  |
| Os06g0704400 | LOC_Os06g49090 | 6:29744427 | G | C | 5_prime_UTR |  |  |  |
| Os06g0704400 | LOC_Os06g49090 | 6:29744789 | A | C | upstream |  |  |  |
| Os06g0704400 | LOC_Os06g49090 | 6:29745305 | T | C | upstream |  |  |  |
| Os06g0704400 | LOC_Os06g49090 | 6:29745441 | A | G | upstream |  |  |  |
| Os06g0704400 | LOC_Os06g49090 | 6:29745482 | G | A | upstream |  |  |  |
| Os06g0704400 | LOC_Os06g49090 | 6:29745509 | C | T | upstream |  |  |  |
| Os06g0704400 | LOC_Os06g49090 | 6:29745518 | G | A | upstream |  |  |  |
| Os06g0704400 | LOC_Os06g49090 | 6:29745593 | C | T | upstream |  |  |  |
| Os06g0704400 | LOC_Os06g49090 | 6:29745830 | A | C | upstream |  |  |  |
| Os06g0704400 | LOC_Os06g49090 | 6:29745935 | A | G | upstream |  |  |  |
| Os06g0704400 | LOC_Os06g49090 | 6:29746113 | T | G | upstream |  |  |  |
| Os06g0704400 | LOC_Os06g49090 | 6:29746125 | A | G | upstream |  |  |  |
| Os06g0704400 | LOC_Os06g49090 | 6:29746331 | T | C | upstream |  |  |  |
| Os06g0704400 | LOC_Os06g49090 | 6:29746621 | G | A | upstream |  |  |  |
| Os06g0704400 | LOC_Os06g49090 | 6:29746899 | G | T | exon | nonsynonymous |  |  |
| Os06g0704400 | LOC_Os06g49090 | 6:29747316 | C | T | exon | synonymous |  |  |
| Os06g0704400 | LOC_Os06g49090 | 6:29747736 | C | T | exon | synonymous |  |  |
| Os06g0704400 | LOC_Os06g49090 | 6:29748292 | T | G | exon | nonsynonymous |  |  |
| Os06g0704400 | LOC_Os06g49090 | 6:29748388 | G | A | exon | nonsynonymous |  |  |
| Os06g0704400 | LOC_Os06g49090 | 6:29748937 | A | G | exon | nonsynonymous |  |  |
| Os06g0704400 | LOC_Os06g49090 | 6:29749906 | T | C | downstream |  |  |  |
| Os06g0704400 | LOC_Os06g49090 | 6:29750423 | A | T | 3_prime_UTR |  |  |  |
| Os06g0704400 | LOC_Os06g49090 | 6:29750430 | A | G | 3_prime_UTR |  |  |  |
| Os06g0704450 | None | 6:29756458 | T | A | upstream |  |  |  |
| Os06g0704450 | None | 6:29756539 | T | A | upstream |  |  |  |
| Os06g0704450 | None | 6:29756546 | A | T | upstream |  |  |  |
| Os06g0704450 | None | 6:29756624 | C | T | upstream |  |  |  |
| Os06g0704450 | None | 6:29756983 | C | T | upstream |  |  |  |
| Os06g0704450 | None | 6:29757130 | A | C | upstream |  |  |  |
| Os06g0704450 | None | 6:29757263 | C | T | upstream |  |  |  |
| Os06g0704450 | None | 6:29758017 | C | T | upstream |  |  |  |
| Os06g0704450 | None | 6:29758199 | G | A | upstream |  |  |  |
| Os06g0704450 | None | 6:29758692 | G | A | upstream |  |  |  |
| Os06g0704450 | None | 6:29759876 | A | G | upstream |  |  |  |
| Os06g0704450 | None | 6:29759887 | G | A | upstream |  |  |  |
| Os06g0704450 | None | 6:29759928 | T | A | upstream |  |  |  |
| Os06g0704500 | LOC_Os06g49100 | 6:29750173 | C | G | upstream |  |  |  |
| Os06g0704500 | LOC_Os06g49100 | 6:29750178 | A | G | upstream |  |  |  |
| Os06g0704500 | LOC_Os06g49100 | 6:29750808 | G | A | upstream |  |  |  |
| Os06g0704500 | LOC_Os06g49100 | 6:29751112 | A | C | upstream |  |  |  |
| Os06g0704500 | LOC_Os06g49100 | 6:29751519 | G | A | upstream |  |  |  |
| Os06g0704500 | LOC_Os06g49100 | 6:29751717 | G | A | upstream |  |  |  |
| Os06g0704500 | LOC_Os06g49100 | 6:29751870 | C | T | upstream |  |  |  |
| Os06g0704500 | LOC_Os06g49100 | 6:29752093 | G | A | upstream |  |  |  |
| Os06g0704500 | LOC_Os06g49100 | 6:29752117 | G | A | upstream |  |  |  |
| Os06g0704500 | LOC_Os06g49100 | 6:29752257 | A | G | upstream |  |  |  |
| Os06g0704500 | LOC_Os06g49100 | 6:29752406 | A | G | upstream |  |  |  |
| Os06g0704500 | LOC_Os06g49100 | 6:29752410 | C | G | upstream |  |  |  |
| Os06g0704500 | LOC_Os06g49100 | 6:29752563 | T | C | upstream |  |  |  |
| Os06g0704500 | LOC_Os06g49100 | 6:29753086 | T | C | upstream |  |  |  |
| Os06g0704500 | LOC_Os06g49100 | 6:29753336 | A | G | upstream |  |  |  |
| Os06g0704500 | LOC_Os06g49100 | 6:29753388 | C | T | upstream |  |  |  |
| Os06g0704500 | LOC_Os06g49100 | 6:29753802 | G | A | upstream |  |  |  |
| Os06g0704500 | LOC_Os06g49100 | 6:29753991 | G | T | upstream |  |  |  |
| Os06g0704500 | LOC_Os06g49100 | 6:29754083 | T | C | upstream |  |  |  |
| Os06g0704500 | LOC_Os06g49100 | 6:29760744 | C | T | downstream |  |  |  |
| Os06g0704600 | LOC_Os06g49110 | 6:29758783 | G | T | intron |  |  |  |
| Os06g0704600 | LOC_Os06g49110 | 6:29761023 | A | T | exon | nonsynonymous |  |  |
| Os06g0704600 | LOC_Os06g49110 | 6:29762839 | G | A | upstream |  |  |  |
| Os06g0704600 | LOC_Os06g49110 | 6:29762973 | G | C | upstream |  |  |  |
| Os06g0704600 | LOC_Os06g49110 | 6:29763292 | C | A | upstream |  |  |  |
| Os06g0704600 | LOC_Os06g49110 | 6:29764160 | A | C | upstream |  |  |  |
| Os06g0704600 | LOC_Os06g49110 | 6:29765535 | T | C | upstream |  |  |  |
| Os06g0704600 | LOC_Os06g49110 | 6:29765569 | G | T | upstream |  |  |  |
| Os06g0704600 | LOC_Os06g49110 | 6:29765578 | A | T | upstream |  |  |  |
| Os06g0704700 | LOC_Os06g49120 | 6:29761570 | C | T | downstream |  |  |  |
| Os06g0704700 | LOC_Os06g49120 | 6:29761616 | G | T | downstream |  |  |  |
| Os06g0704700 | LOC_Os06g49120 | 6:29761632 | A | T | downstream |  |  |  |
| Os06g0704700 | LOC_Os06g49120 | 6:29762555 | C | T | 3_prime_UTR |  |  |  |
| Os06g0704700 | LOC_Os06g49120 | 6:29762938 | T | C | exon | nonsynonymous |  |  |
| Os06g0704700 | LOC_Os06g49120 | 6:29764303 | A | T | exon | synonymous |  |  |
| Os06g0704700 | LOC_Os06g49120 | 6:29766993 | T | C | upstream |  |  |  |
| Os06g0704700 | LOC_Os06g49120 | 6:29767120 | C | T | upstream |  |  |  |
| Os06g0704700 | LOC_Os06g49120 | 6:29767191 | T | C | upstream |  |  |  |
| Os06g0704700 | LOC_Os06g49120 | 6:29767204 | G | A | upstream |  |  |  |
| Os06g0704700 | LOC_Os06g49120 | 6:29767328 | G | A | upstream |  |  |  |
| Os06g0704700 | LOC_Os06g49120 | 6:29768440 | G | T | upstream |  |  |  |
| Os06g0704700 | LOC_Os06g49120 | 6:29768798 | T | A | upstream |  |  |  |
| Os06g0704700 | LOC_Os06g49120 | 6:29769233 | G | C | upstream |  |  |  |
| Os06g0704700 | LOC_Os06g49120 | 6:29769260 | T | A | upstream |  |  |  |
| Os06g0704700 | LOC_Os06g49120 | 6:29769660 | C | T | upstream |  |  |  |
| Os06g0704700 | LOC_Os06g49120 | 6:29770452 | C | T | upstream |  |  |  |
| Os06g0704700 | LOC_Os06g49120 | 6:29770478 | T | C | upstream |  |  |  |
| Os06g0704700 | LOC_Os06g49120 | 6:29771499 | T | C | upstream |  |  |  |
| Os06g0704700 | LOC_Os06g49120 | 6:29771647 | A | C | upstream |  |  |  |
| Os06g0704800 | LOC_Os06g49130 | 6:29769334 | C | A | exon | nonsynonymous |  |  |
| Os06g0704800 | LOC_Os06g49130 | 6:29769718 | T | C | exon | nonsynonymous |  |  |
| Os06g0704800 | LOC_Os06g49130 | 6:29770917 | A | G | exon | nonsynonymous |  |  |
| Os06g0704800 | LOC_Os06g49130 | 6:29771012 | G | A | exon | nonsynonymous |  |  |
| Os06g0704800 | LOC_Os06g49130 | 6:29771360 | C | A | exon | nonsynonymous |  |  |
| Os06g0704800 | LOC_Os06g49130 | 6:29771834 | T | C | exon | nonsynonymous |  |  |
| Os06g0704800 | LOC_Os06g49130 | 6:29772154 | C | T | exon | nonsynonymous |  |  |
| Os06g0704800 | LOC_Os06g49130 | 6:29772412 | T | C | exon | nonsynonymous |  |  |
| Os06g0704800 | LOC_Os06g49130 | 6:29772629 | A | G | intron |  |  |  |
| Os06g0704800 | LOC_Os06g49130 | 6:29772889 | C | T | exon | nonsynonymous |  |  |
| Os06g0704800 | LOC_Os06g49130 | 6:29773351 | T | C | intron |  |  |  |
| Os06g0704800 | LOC_Os06g49130 | 6:29773670 | G | A | intron |  |  |  |
| Os06g0704800 | LOC_Os06g49130 | 6:29773695 | G | A | intron |  |  |  |
| Os06g0704800 | LOC_Os06g49130 | 6:29773873 | A | G | intron |  |  |  |
| Os06g0704800 | LOC_Os06g49130 | 6:29773884 | C | T | intron |  |  |  |
| Os06g0704800 | LOC_Os06g49130 | 6:29773960 | T | C | exon | nonsynonymous |  |  |
| Os06g0704800 | LOC_Os06g49130 | 6:29774085 | A | G | exon | nonsynonymous |  |  |
| Os06g0704800 | LOC_Os06g49130 | 6:29774249 | G | A | exon | synonymous |  |  |
| Os06g0704800 | LOC_Os06g49130 | 6:29774647 | A | G | exon | nonsynonymous |  |  |
| Os06g0704800 | LOC_Os06g49130 | 6:29775844 | G | A | intron |  |  |  |
| Os06g0704800 | LOC_Os06g49130 | 6:29776038 | G | T | intron |  |  |  |
| Os06g0704800 | LOC_Os06g49130 | 6:29776228 | G | A | intron |  |  |  |
| Os06g0704800 | LOC_Os06g49130 | 6:29776248 | A | G | intron |  |  |  |
| Os06g0704800 | LOC_Os06g49130 | 6:29776452 | A | G | intron |  |  |  |
| Os06g0704800 | LOC_Os06g49130 | 6:29776648 | A | G | intron |  |  |  |
| Os06g0704800 | LOC_Os06g49130 | 6:29776859 | C | T | intron |  |  |  |
| Os06g0704800 | LOC_Os06g49130 | 6:29780518 | C | A | exon | nonsynonymous |  |  |
| Os06g0704800 | LOC_Os06g49130 | 6:29780566 | A | C | exon | nonsynonymous |  |  |
| Os06g0704800 | LOC_Os06g49130 | 6:29781078 | G | C | upstream |  |  |  |
| Os06g0704800 | LOC_Os06g49130 | 6:29781106 | G | T | upstream |  |  |  |
| Os06g0704800 | LOC_Os06g49130 | 6:29781117 | C | G | upstream |  |  |  |
| Os06g0704800 | LOC_Os06g49130 | 6:29781118 | C | T | upstream |  |  |  |
| Os06g0704800 | LOC_Os06g49130 | 6:29781151 | G | A | upstream |  |  |  |
| Os06g0704800 | LOC_Os06g49130 | 6:29781217 | C | T | upstream |  |  |  |
| Os06g0704800 | LOC_Os06g49130 | 6:29781231 | G | A | upstream |  |  |  |
| Os06g0704800 | LOC_Os06g49130 | 6:29781391 | A | T | upstream |  |  |  |
| Os06g0704800 | LOC_Os06g49130 | 6:29781473 | T | C | upstream |  |  |  |
| Os06g0704800 | LOC_Os06g49130 | 6:29781483 | A | T | upstream |  |  |  |
| Os06g0704800 | LOC_Os06g49130 | 6:29781531 | T | C | upstream |  |  |  |
| Os06g0704800 | LOC_Os06g49130 | 6:29781686 | G | A | upstream |  |  |  |
| Os06g0704800 | LOC_Os06g49130 | 6:29781929 | T | C | upstream |  |  |  |
| Os06g0704800 | LOC_Os06g49130 | 6:29782339 | G | C | upstream |  |  |  |
| Os06g0704800 | LOC_Os06g49130 | 6:29783004 | T | G | upstream |  |  |  |
| Os06g0704800 | LOC_Os06g49130 | 6:29783263 | C | T | upstream |  |  |  |
| Os06g0704800 | LOC_Os06g49130 | 6:29783470 | T | A | upstream |  |  |  |
| Os06g0704800 | LOC_Os06g49130 | 6:29783717 | C | T | upstream |  |  |  |
| Os06g0704800 | LOC_Os06g49130 | 6:29784118 | C | T | upstream |  |  |  |
| Os06g0704800 | LOC_Os06g49130 | 6:29784982 | A | C | upstream |  |  |  |
| Os06g0704800 | LOC_Os06g49130 | 6:29785060 | G | T | upstream |  |  |  |
| Os06g0704800 | LOC_Os06g49130 | 6:29785103 | G | T | upstream |  |  |  |
| Os06g0704800 | LOC_Os06g49130 | 6:29785397 | T | C | upstream |  |  |  |
| Os06g0704800 | LOC_Os06g49130 | 6:29785425 | C | T | upstream |  |  |  |
| Os06g0704800 | LOC_Os06g49130 | 6:29785541 | C | A | upstream |  |  |  |
| Os06g0704800 | LOC_Os06g49130 | 6:29785544 | C | T | upstream |  |  |  |
| Os06g0704800 | LOC_Os06g49130 | 6:29785645 | C | T | upstream |  |  |  |
| Os06g0704800 | LOC_Os06g49130 | 6:29785650 | T | C | upstream |  |  |  |
| Os06g0704900 | LOC_Os06g49140 | 6:29777734 | A | T | upstream |  |  |  |
| Os06g0704900 | LOC_Os06g49140 | 6:29778586 | C | T | upstream |  |  |  |
| Os06g0704900 | LOC_Os06g49140 | 6:29778593 | C | T | upstream |  |  |  |
| Os06g0704900 | LOC_Os06g49140 | 6:29778696 | G | A | upstream |  |  |  |
| Os06g0704900 | LOC_Os06g49140 | 6:29778780 | A | T | upstream |  |  |  |
| Os06g0704900 | LOC_Os06g49140 | 6:29778889 | T | C | upstream |  |  |  |
| Os06g0704900 | LOC_Os06g49140 | 6:29779162 | G | A | upstream |  |  |  |
| Os06g0704900 | LOC_Os06g49140 | 6:29779381 | T | C | upstream |  |  |  |
| Os06g0704900 | LOC_Os06g49140 | 6:29779713 | G | A | upstream |  |  |  |
| Os06g0704900 | LOC_Os06g49140 | 6:29779941 | G | A | upstream |  |  |  |
| Os06g0704900 | LOC_Os06g49140 | 6:29780137 | A | G | upstream |  |  |  |
| Os06g0704900 | LOC_Os06g49140 | 6:29780416 | C | T | upstream |  |  |  |
| Os06g0704900 | LOC_Os06g49140 | 6:29782226 | T | C | exon | synonymous |  |  |
| Os06g0704900 | LOC_Os06g49140 | 6:29782710 | G | A | exon | synonymous |  |  |
| Os06g0704900 | LOC_Os06g49140 | 6:29784514 | T | G | exon | nonsynonymous |  |  |
| Os06g0704900 | LOC_Os06g49140 | 6:29784523 | C | T | exon | nonsynonymous |  |  |
| Os06g0705000 | LOC_Os06g49150 | 6:29785888 | T | A | upstream |  |  |  |
| Os06g0705000 | LOC_Os06g49150 | 6:29786115 | C | A | upstream |  |  |  |
| Os06g0705000 | LOC_Os06g49150 | 6:29787655 | T | A | exon | nonsynonymous |  |  |
| Os06g0705000 | LOC_Os06g49150 | 6:29788420 | C | G | exon | nonsynonymous |  |  |
| Os06g0705000 | LOC_Os06g49150 | 6:29788439 | C | T | exon | synonymous |  |  |
| Os06g0705000 | LOC_Os06g49150 | 6:29792431 | T | G | downstream |  |  |  |
| Os06g0705000 | LOC_Os06g49150 | 6:29793528 | A | G | downstream |  |  |  |
| Os06g0705100 | LOC_Os06g49160 | 6:29787824 | T | C | upstream |  |  |  |
| Os06g0705100 | LOC_Os06g49160 | 6:29787831 | T | C | upstream |  |  |  |
| Os06g0705100 | LOC_Os06g49160 | 6:29787835 | T | G | upstream |  |  |  |
| Os06g0705100 | LOC_Os06g49160 | 6:29788733 | C | A | upstream |  |  |  |
| Os06g0705100 | LOC_Os06g49160 | 6:29788940 | T | C | upstream |  |  |  |
| Os06g0705100 | LOC_Os06g49160 | 6:29789500 | T | C | exon | synonymous |  |  |
| Os06g0705100 | LOC_Os06g49160 | 6:29790496 | T | G | 3_prime_UTR |  |  |  |
| Os06g0705100 | LOC_Os06g49160 | 6:29790547 | T | C | 3_prime_UTR |  |  |  |
| Os06g0705100 | LOC_Os06g49160 | 6:29793853 | T | C | downstream |  |  |  |
| Os06g0705100 | LOC_Os06g49160 | 6:29793918 | A | C | downstream |  |  |  |
| Os06g0705100 | LOC_Os06g49160 | 6:29793926 | T | C | downstream |  |  |  |
| Os06g0705100 | LOC_Os06g49160 | 6:29794193 | C | G | downstream |  |  |  |
| Os06g0705100 | LOC_Os06g49160 | 6:29794223 | C | T | downstream |  |  |  |
| Os06g0705100 | LOC_Os06g49160 | 6:29794296 | G | A | downstream |  |  |  |
| Os06g0705100 | LOC_Os06g49160 | 6:29794305 | A | G | downstream |  |  |  |
| Os06g0705200 | LOC_Os06g49170 | 6:29792222 | C | T | exon | synonymous |  |  |
| Os06g0705200 | LOC_Os06g49170 | 6:29792267 | G | T | exon | synonymous |  |  |
| Os06g0705200 | LOC_Os06g49170 | 6:29794953 | G | T | exon | nonsynonymous |  |  |
| Os06g0705200 | LOC_Os06g49170 | 6:29795509 | C | T | upstream |  |  |  |
| Os06g0705200 | LOC_Os06g49170 | 6:29795692 | T | A | upstream |  |  |  |
| Os06g0705200 | LOC_Os06g49170 | 6:29795710 | C | A | upstream |  |  |  |
| Os06g0705200 | LOC_Os06g49170 | 6:29795763 | T | C | upstream |  |  |  |
| Os06g0705200 | LOC_Os06g49170 | 6:29795796 | T | G | upstream |  |  |  |
| Os06g0705200 | LOC_Os06g49170 | 6:29795839 | G | A | upstream |  |  |  |
| Os06g0705200 | LOC_Os06g49170 | 6:29795893 | A | G | upstream |  |  |  |
| Os06g0705200 | LOC_Os06g49170 | 6:29795943 | T | C | upstream |  |  |  |
| Os06g0705200 | LOC_Os06g49170 | 6:29796970 | T | A | upstream |  |  |  |
| Os06g0705200 | LOC_Os06g49170 | 6:29797017 | T | C | upstream |  |  |  |
| Os06g0705200 | LOC_Os06g49170 | 6:29797117 | G | A | upstream |  |  |  |
| Os06g0705200 | LOC_Os06g49170 | 6:29797361 | A | G | upstream |  |  |  |
| Os06g0705200 | LOC_Os06g49170 | 6:29797590 | A | G | upstream |  |  |  |
| Os06g0705200 | LOC_Os06g49170 | 6:29797591 | T | A | upstream |  |  |  |
| Os06g0705200 | LOC_Os06g49170 | 6:29797618 | T | C | upstream |  |  |  |
| Os06g0705200 | LOC_Os06g49170 | 6:29797768 | T | C | upstream |  |  |  |
| Os06g0705200 | LOC_Os06g49170 | 6:29797810 | C | T | upstream |  |  |  |
| Os06g0705200 | LOC_Os06g49170 | 6:29797859 | G | T | upstream |  |  |  |
| Os06g0705200 | LOC_Os06g49170 | 6:29797885 | G | A | upstream |  |  |  |
| Os06g0705200 | LOC_Os06g49170 | 6:29797893 | C | T | upstream |  |  |  |
| Os06g0705200 | LOC_Os06g49170 | 6:29797953 | G | A | upstream |  |  |  |
| Os06g0705200 | LOC_Os06g49170 | 6:29798611 | T | G | upstream |  |  |  |
| Os06g0705200 | LOC_Os06g49170 | 6:29798756 | T | A | upstream |  |  |  |
| Os06g0705250 | None | 6:29790121 | T | C | upstream |  |  |  |
| Os06g0705250 | None | 6:29790166 | C | T | upstream |  |  |  |
| Os06g0705250 | None | 6:29791119 | G | A | upstream |  |  |  |
| Os06g0705250 | None | 6:29791131 | A | G | upstream |  |  |  |
| Os06g0705300 | LOC_Os06g49185 | 6:29798185 | C | A | 3_prime_UTR |  |  |  |
| Os06g0705300 | LOC_Os06g49185 | 6:29798244 | G | T | 3_prime_UTR |  |  |  |
| Os06g0705300 | LOC_Os06g49185 | 6:29798685 | G | T | exon | nonsynonymous |  |  |
| Os06g0705300 | LOC_Os06g49185 | 6:29799186 | G | A | exon | nonsynonymous |  |  |
| Os06g0705300 | LOC_Os06g49185 | 6:29799691 | T | A | exon | nonsynonymous |  |  |
| Os06g0705300 | LOC_Os06g49185 | 6:29799945 | A | G | 3_prime_UTR |  |  |  |
| Os06g0705300 | LOC_Os06g49185 | 6:29800054 | A | G | 3_prime_UTR |  |  |  |
| Os06g0705300 | LOC_Os06g49185 | 6:29800102 | A | T | 3_prime_UTR |  |  |  |
| Os06g0705300 | LOC_Os06g49185 | 6:29801023 | G | C | 5_prime_UTR |  |  |  |
| Os06g0705300 | LOC_Os06g49185 | 6:29801430 | A | T | 3_prime_UTR |  |  |  |
| Os06g0705300 | LOC_Os06g49185 | 6:29801781 | C | T | 3_prime_UTR |  |  |  |
| Os06g0705300 | LOC_Os06g49185 | 6:29801791 | T | C | 3_prime_UTR |  |  |  |
| Os06g0705300 | LOC_Os06g49185 | 6:29802483 | C | T | exon | synonymous |  |  |
| Os06g0705300 | LOC_Os06g49185 | 6:29802875 | C | G | exon | nonsynonymous |  |  |
| Os06g0705300 | LOC_Os06g49185 | 6:29802894 | C | A | exon | synonymous |  |  |
| Os06g0705300 | LOC_Os06g49185 | 6:29803052 | C | A | exon | nonsynonymous |  |  |
| Os06g0705300 | LOC_Os06g49185 | 6:29803112 | C | T | exon | nonsynonymous |  |  |
| Os06g0705300 | LOC_Os06g49185 | 6:29803470 | T | C | exon | synonymous |  |  |
| Os06g0705300 | LOC_Os06g49185 | 6:29803562 | C | A | exon | nonsynonymous |  |  |
| Os06g0705300 | LOC_Os06g49185 | 6:29804437 | G | A | upstream |  |  |  |
| Os06g0705300 | LOC_Os06g49185 | 6:29804467 | T | A | upstream |  |  |  |
| Os06g0705300 | LOC_Os06g49185 | 6:29804493 | T | C | upstream |  |  |  |
| Os06g0705300 | LOC_Os06g49185 | 6:29807337 | T | A | upstream |  |  |  |
| Os06g0705300 | LOC_Os06g49185 | 6:29807345 | C | T | upstream |  |  |  |
| Os06g0705300 | LOC_Os06g49185 | 6:29807460 | C | T | upstream |  |  |  |
| Os06g0705300 | LOC_Os06g49185 | 6:29807585 | G | A | upstream |  |  |  |
| Os06g0705300 | LOC_Os06g49185 | 6:29808501 | T | C | upstream |  |  |  |
| Os06g0705350 | None | 6:29800470 | G | C | downstream |  |  |  |
| Os06g0705350 | None | 6:29804936 | G | A | exon | synonymous |  |  |
| Os06g0705350 | None | 6:29806140 | A | G | exon | nonsynonymous |  |  |
| Os06g0705350 | None | 6:29806679 | G | C | exon | synonymous |  |  |
| Os06g0705350 | None | 6:29810118 | G | A | upstream |  |  |  |
| Os06g0705350 | None | 6:29810221 | C | T | upstream |  |  |  |
| Os06g0705350 | None | 6:29810412 | A | C | upstream |  |  |  |
| Os06g0705350 | None | 6:29810498 | C | A | upstream |  |  |  |
| Os06g0705350 | None | 6:29810708 | G | C | upstream |  |  |  |
| Os06g0705350 | None | 6:29810827 | T | G | upstream |  |  |  |
| Os06g0705350 | None | 6:29811252 | G | A | upstream |  |  |  |
| Os06g0705350 | None | 6:29811294 | T | G | upstream |  |  |  |
| Os06g0705350 | None | 6:29811441 | C | G | upstream |  |  |  |
| Os06g0705350 | None | 6:29811492 | T | C | upstream |  |  |  |
| Os06g0705350 | None | 6:29811980 | C | G | upstream |  |  |  |
| Os06g0705350 | None | 6:29811981 | T | C | upstream |  |  |  |
| Os06g0705400 | LOC_Os06g49190 | 6:29808364 | T | C | 3_prime_UTR |  |  |  |
| Os06g0705400 | LOC_Os06g49190 | 6:29808412 | T | A | 3_prime_UTR |  |  |  |
| Os06g0705500 | LOC_Os06g49200 | 6:29809826 | G | C | exon | nonsynonymous |  |  |
| Os06g0705500 | LOC_Os06g49200 | 6:29809865 | C | T | exon | nonsynonymous |  |  |
| Os06g0705500 | LOC_Os06g49200 | 6:29811735 | G | C | 5_prime_UTR |  |  |  |
| Os06g0705500 | LOC_Os06g49200 | 6:29812227 | A | G | upstream |  |  |  |
| Os06g0705500 | LOC_Os06g49200 | 6:29812306 | A | G | upstream |  |  |  |
| Os06g0705500 | LOC_Os06g49200 | 6:29812325 | G | A | upstream |  |  |  |
| Os06g0705500 | LOC_Os06g49200 | 6:29812414 | C | T | upstream |  |  |  |
| Os06g0705500 | LOC_Os06g49200 | 6:29812416 | T | A | upstream |  |  |  |
| Os06g0705500 | LOC_Os06g49200 | 6:29812418 | T | C | upstream |  |  |  |
| Os06g0705500 | LOC_Os06g49200 | 6:29812535 | A | G | upstream |  |  |  |
| Os06g0705500 | LOC_Os06g49200 | 6:29812581 | A | C | upstream |  |  |  |
| Os06g0705500 | LOC_Os06g49200 | 6:29812648 | G | A | upstream |  |  |  |
| Os06g0705500 | LOC_Os06g49200 | 6:29812725 | A | G | upstream |  |  |  |
| Os06g0705500 | LOC_Os06g49200 | 6:29813168 | G | A | upstream |  |  |  |
| Os06g0705500 | LOC_Os06g49200 | 6:29813175 | G | A | upstream |  |  |  |
| Os06g0705500 | LOC_Os06g49200 | 6:29813247 | A | G | upstream |  |  |  |
| Os06g0705500 | LOC_Os06g49200 | 6:29813286 | C | T | upstream |  |  |  |
| Os06g0705500 | LOC_Os06g49200 | 6:29813456 | T | A | upstream |  |  |  |
| Os06g0705500 | LOC_Os06g49200 | 6:29813603 | A | G | upstream |  |  |  |
| Os06g0705500 | LOC_Os06g49200 | 6:29814023 | G | A | upstream |  |  |  |
| Os06g0705500 | LOC_Os06g49200 | 6:29814381 | A | C | upstream |  |  |  |
| Os06g0705500 | LOC_Os06g49200 | 6:29814687 | A | C | upstream |  |  |  |
| Os06g0705500 | LOC_Os06g49200 | 6:29815483 | T | C | upstream |  |  |  |
| Os06g0705651 | None | 6:29816915 | T | C | upstream |  |  |  |
| Os06g0705651 | None | 6:29817106 | A | T | upstream |  |  |  |
| Os06g0705651 | None | 6:29817250 | A | G | upstream |  |  |  |
| Os06g0705651 | None | 6:29817378 | T | C | upstream |  |  |  |
| Os06g0705651 | None | 6:29817484 | G | A | upstream |  |  |  |
| Os06g0705651 | None | 6:29817549 | T | G | upstream |  |  |  |
| Os06g0705651 | None | 6:29817662 | T | G | upstream |  |  |  |
| Os06g0705651 | None | 6:29817703 | G | A | upstream |  |  |  |
| Os06g0705651 | None | 6:29817754 | G | C | upstream |  |  |  |
| Os06g0705651 | None | 6:29817808 | G | A | upstream |  |  |  |
| Os06g0705651 | None | 6:29818127 | T | C | upstream |  |  |  |
| Os06g0705651 | None | 6:29818173 | A | T | upstream |  |  |  |
| Os06g0705651 | None | 6:29818214 | C | T | upstream |  |  |  |
| Os06g0705651 | None | 6:29818371 | C | T | upstream |  |  |  |
| Os06g0705651 | None | 6:29818478 | T | C | upstream |  |  |  |
| Os06g0705651 | None | 6:29818569 | A | G | upstream |  |  |  |
| Os06g0705651 | None | 6:29818650 | C | T | upstream |  |  |  |
| Os06g0705651 | None | 6:29818737 | C | T | upstream |  |  |  |
| Os06g0705651 | None | 6:29818774 | T | A | upstream |  |  |  |
| Os06g0705651 | None | 6:29818929 | G | A | upstream |  |  |  |
| Os06g0705651 | None | 6:29818959 | T | G | upstream |  |  |  |
| Os06g0705651 | None | 6:29819055 | C | A | upstream |  |  |  |
| Os06g0705651 | None | 6:29819209 | C | T | upstream |  |  |  |
| Os06g0705651 | None | 6:29819297 | A | G | upstream |  |  |  |
| Os06g0705651 | None | 6:29819311 | G | A | upstream |  |  |  |
| Os06g0705651 | None | 6:29820589 | T | C | upstream |  |  |  |
| Os06g0705651 | None | 6:29820725 | G | A | upstream |  |  |  |
| Os06g0705651 | None | 6:29820777 | T | C | upstream |  |  |  |
| Os06g0705651 | None | 6:29820852 | T | C | upstream |  |  |  |
| Os06g0705651 | None | 6:29820913 | C | T | upstream |  |  |  |
| Os06g0705651 | None | 6:29820917 | C | T | upstream |  |  |  |
| Os06g0705651 | None | 6:29821348 | G | C | upstream |  |  |  |
| Os06g0705651 | None | 6:29821405 | T | A | upstream |  |  |  |
| Os06g0705700 | LOC_Os06g49220 | 6:29821951 | C | T | upstream |  |  |  |
| Os06g0705700 | LOC_Os06g49220 | 6:29821985 | G | A | upstream |  |  |  |
| Os06g0705700 | LOC_Os06g49220 | 6:29822041 | A | G | upstream |  |  |  |
| Os06g0705700 | LOC_Os06g49220 | 6:29822164 | T | A | upstream |  |  |  |
| Os06g0705700 | LOC_Os06g49220 | 6:29822166 | T | A | upstream |  |  |  |
| Os06g0705700 | LOC_Os06g49220 | 6:29822550 | C | G | upstream |  |  |  |
| Os06g0705700 | LOC_Os06g49220 | 6:29822678 | T | C | upstream |  |  |  |
| Os06g0705700 | LOC_Os06g49220 | 6:29822699 | A | T | upstream |  |  |  |
| Os06g0705700 | LOC_Os06g49220 | 6:29822996 | T | C | upstream |  |  |  |
| Os06g0705700 | LOC_Os06g49220 | 6:29823408 | G | A | upstream |  |  |  |
| Os06g0705700 | LOC_Os06g49220 | 6:29823499 | C | T | upstream |  |  |  |
| Os06g0705700 | LOC_Os06g49220 | 6:29823729 | G | A | upstream |  |  |  |
| Os06g0705700 | LOC_Os06g49220 | 6:29823731 | G | A | upstream |  |  |  |
| Os06g0705700 | LOC_Os06g49220 | 6:29823802 | A | C | upstream |  |  |  |
| Os06g0705700 | LOC_Os06g49220 | 6:29824026 | C | T | upstream |  |  |  |
| Os06g0705700 | LOC_Os06g49220 | 6:29824343 | G | A | upstream |  |  |  |
| Os06g0705700 | LOC_Os06g49220 | 6:29824473 | C | G | exon | nonsynonymous |  |  |
| Os06g0705700 | LOC_Os06g49220 | 6:29824475 | C | A | exon | nonsynonymous |  |  |
| Os06g0705901 | None | 6:29826486 | T | G | exon | nonsynonymous |  |  |
| Os06g0705901 | None | 6:29826736 | C | T | upstream |  |  |  |
| Os06g0705901 | None | 6:29826745 | G | A | upstream |  |  |  |
| Os06g0705901 | None | 6:29826825 | G | T | upstream |  |  |  |
| Os06g0705901 | None | 6:29826987 | C | T | upstream |  |  |  |
| Os06g0705901 | None | 6:29827071 | T | C | upstream |  |  |  |
| Os06g0705901 | None | 6:29827098 | G | A | upstream |  |  |  |
| Os06g0705901 | None | 6:29827380 | G | T | upstream |  |  |  |
| Os06g0705901 | None | 6:29827401 | G | A | upstream |  |  |  |
| Os06g0705901 | None | 6:29827585 | A | G | upstream |  |  |  |
| Os06g0705901 | None | 6:29827597 | G | A | upstream |  |  |  |
| Os06g0705901 | None | 6:29827651 | C | T | upstream |  |  |  |
| Os06g0705901 | None | 6:29827864 | G | A | upstream |  |  |  |
| Os06g0705901 | None | 6:29827879 | T | G | upstream |  |  |  |
| Os06g0705901 | None | 6:29828052 | C | T | upstream |  |  |  |
| Os06g0705901 | None | 6:29828236 | T | G | upstream |  |  |  |
| Os06g0705901 | None | 6:29828442 | G | A | upstream |  |  |  |
| Os06g0705901 | None | 6:29828467 | G | T | upstream |  |  |  |
| Os06g0705901 | None | 6:29828494 | C | T | upstream |  |  |  |
| Os06g0705901 | None | 6:29828782 | A | C | upstream |  |  |  |
| Os06g0705901 | None | 6:29828783 | T | A | upstream |  |  |  |
| Os06g0705901 | None | 6:29829058 | T | C | upstream |  |  |  |
| Os06g0705901 | None | 6:29829113 | T | C | upstream |  |  |  |
| Os06g0705901 | None | 6:29829188 | G | A | upstream |  |  |  |
| Os06g0705901 | None | 6:29830046 | T | C | upstream |  |  |  |
| Os06g0705901 | None | 6:29831371 | A | C | upstream |  |  |  |
| Os06g0706000 | None | 6:29835940 | T | G | upstream |  |  |  |
| Os06g0706000 | None | 6:29835948 | C | T | upstream |  |  |  |
| Os06g0706000 | None | 6:29836002 | G | T | upstream |  |  |  |
| Os06g0706000 | None | 6:29836039 | G | A | upstream |  |  |  |
| Os06g0706100 | LOC_Os06g49240 | 6:29824543 | C | G | upstream |  |  |  |
| Os06g0706400 | LOC_Os06g49250 | 6:29836313 | G | A | upstream |  |  |  |
| Os06g0706400 | LOC_Os06g49250 | 6:29836340 | G | A | upstream |  |  |  |
| Os06g0706400 | LOC_Os06g49250 | 6:29836347 | C | T | upstream |  |  |  |
| Os06g0706400 | LOC_Os06g49250 | 6:29836506 | G | A | upstream |  |  |  |
| Os06g0706400 | LOC_Os06g49250 | 6:29836668 | C | A | upstream |  |  |  |
| Os06g0706400 | LOC_Os06g49250 | 6:29836739 | G | T | upstream |  |  |  |
| Os06g0706400 | LOC_Os06g49250 | 6:29837086 | C | T | upstream |  |  |  |
| Os06g0706400 | LOC_Os06g49250 | 6:29837187 | G | A | upstream |  |  |  |
| Os06g0706400 | LOC_Os06g49250 | 6:29837243 | T | C | upstream |  |  |  |
| Os06g0706400 | LOC_Os06g49250 | 6:29837264 | C | T | upstream |  |  |  |
| Os06g0706400 | LOC_Os06g49250 | 6:29837278 | A | T | upstream |  |  |  |
| Os06g0706400 | LOC_Os06g49250 | 6:29837310 | C | A | upstream |  |  |  |
| Os06g0706400 | LOC_Os06g49250 | 6:29837351 | T | G | upstream |  |  |  |
| Os06g0706400 | LOC_Os06g49250 | 6:29837425 | G | A | upstream |  |  |  |
| Os06g0706400 | LOC_Os06g49250 | 6:29837426 | T | C | upstream |  |  |  |
| Os06g0706400 | LOC_Os06g49250 | 6:29837500 | A | C | upstream |  |  |  |
| Os06g0706400 | LOC_Os06g49250 | 6:29837751 | A | C | upstream |  |  |  |
| Os06g0706400 | LOC_Os06g49250 | 6:29837797 | C | A | upstream |  |  |  |
| Os06g0706400 | LOC_Os06g49250 | 6:29837973 | G | T | upstream |  |  |  |
| Os06g0706400 | LOC_Os06g49250 | 6:29837975 | A | T | upstream |  |  |  |
| Os06g0706400 | LOC_Os06g49250 | 6:29839538 | G | A | 5_prime_UTR |  |  |  |
| Os06g0706400 | LOC_Os06g49250 | 6:29839547 | G | A | 5_prime_UTR |  |  |  |
| Os06g0706400 | LOC_Os06g49250 | 6:29839642 | C | T | 5_prime_UTR |  |  |  |
| Os06g0706400 | LOC_Os06g49250 | 6:29839744 | T | A | exon | nonsynonymous |  |  |
| Os06g0706400 | LOC_Os06g49250 | 6:29841078 | G | A | 3_prime_UTR |  |  |  |
| Os06g0706400 | LOC_Os06g49250 | 6:29841788 | A | G | downstream |  |  |  |
| Os06g0706400 | LOC_Os06g49250 | 6:29843706 | G | A | downstream |  |  |  |
| Os06g0706400 | LOC_Os06g49250 | 6:29843761 | C | T | downstream |  |  |  |
| Os06g0706500 | LOC_Os06g49250 | 6:29838441 | A | T | upstream |  |  |  |
| Os06g0706500 | LOC_Os06g49250 | 6:29838451 | T | C | upstream |  |  |  |
| Os06g0706500 | LOC_Os06g49250 | 6:29838756 | T | A | upstream |  |  |  |
| Os06g0706500 | LOC_Os06g49250 | 6:29838902 | A | T | upstream |  |  |  |
| Os06g0706500 | LOC_Os06g49250 | 6:29838903 | A | C | upstream |  |  |  |
| Os06g0706500 | LOC_Os06g49250 | 6:29841393 | T | C | upstream |  |  |  |
| Os06g0706500 | LOC_Os06g49250 | 6:29841437 | C | T | exon | synonymous |  |  |
| Os06g0706500 | LOC_Os06g49250 | 6:29841450 | A | G | exon | nonsynonymous |  |  |
| Os06g0706600 | LOC_Os06g49260 | 6:29849004 | G | A | downstream |  |  |  |
| Os06g0706600 | LOC_Os06g49260 | 6:29849177 | T | C | downstream |  |  |  |
| Os06g0706600 | LOC_Os06g49260 | 6:29849201 | T | C | downstream |  |  |  |
| Os06g0706600 | LOC_Os06g49260 | 6:29849275 | G | A | downstream |  |  |  |
| Os06g0706600 | LOC_Os06g49260 | 6:29849284 | T | C | downstream |  |  |  |
| Os06g0706600 | LOC_Os06g49260 | 6:29849291 | T | C | downstream |  |  |  |
| Os06g0706600 | LOC_Os06g49260 | 6:29849353 | C | A | downstream |  |  |  |
| Os06g0706600 | LOC_Os06g49260 | 6:29849500 | G | T | downstream |  |  |  |
| Os06g0706600 | LOC_Os06g49260 | 6:29849658 | T | C | downstream |  |  |  |
| Os06g0706600 | LOC_Os06g49260 | 6:29849667 | T | C | downstream |  |  |  |
