# Supplementary material for: An Analysis of Natural Variation Reveals That OsFLA2 Controls Flag Leaf Angle in Rice (Oryza sativa L.)
Source: Front Plant Sci. 2022 Jun 23;13:906912. doi: 10.3389/fpls.2022.906912 (PMC9260283; doi:10.3389/fpls.2022.906912)
Supplement: Supplementary Table 5 — Candidate gene annotation in the region 29.63–29.83 Mb associated with FLA. [file Table_5.DOC]

**Table S5.** Candidate gene annotation in the region 29.63-29.83 Mb associated with flag leaf angle.

| Number | Gene ID | MSU ID | Position | Annotation |
| --- | --- | --- | --- | --- |
| 1 | Os06g0702500 | LOC_Os06g48940 | 29,626,275-29,634,668 | GHMP kinases ATP-binding protein |
| 2 | Os06g0702600 | LOC_Os06g48950 | 29,657,101-29,665,060 | auxin response factor 19, control of leaf angle |
| 3 | Os06g0702700 | LOC_Os06g48960 | 29,665,362-29,667,372 | AIG2-like family domain containing protein |
| 4 | Os06g0702800 | LOC_Os06g48970 | 29,670,439-29,673,498 | expressed protein |
| 5 | Os06g0703000 | LOC_Os06g48980 | 29,675,121-29,678,081 | protein kinase APK1B, chloroplast precursor |
| 6 | Os06g0703200 | LOC_Os06g48990 | 29,679,418-29,682,499 | leucine Rich Repeat family protein |
| 7 | Os06g0703300 | LOC_Os06g49000 | 29,696,560-29,703,207 | expressed protein |
| 8 | Os06g0703400 | None | 29,700,925-29,702,215 | hypothetical protein |
| 9 | Os06g0703500 | LOC_Os06g49010 | 29,703,558-29,708,687 | OsSPL12 - SBP-box gene family member |
| 10 | Os06g0703550 | None | 29,703,852-29,704,214 | hypothetical protein |
| 11 | Os06g0703600 | LOC_Os06g49020 | 29,711,342-29,712,615 | 26S proteasome non-ATPase regulatory subunit 14 |
| 12 | Os06g0703700 | None | 29,711,579-29,712,715 | hypothetical protein |
| 13 | Os06g0703800 | LOC_Os06g49030 | 29,713,469-29,716,973 | activator of 90 kDa heat shock protein ATPase homolog |
| 14 | Os06g0703900 | LOC_Os06g49040 | 29,723,508-29,727,074 | MYB-CC domain containing transcription factor |
| 15 | Os06g0704000 | LOC_Os06g49050 | 29,727,335-29,731,920 | hAT dimerisation domain containing protein |
| 16 | Os06g0704100 | LOC_Os06g49060 | 29,733,805-29,737,188 | ternary complex factor MIP1 |
| 17 | Os06g0704200 | LOC_Os06g49070 | 29,737,266-29,738,301 | expressed protein |
| 18 | Os06g0704300 | LOC_Os06g49080 | 29,738,880-29,742,499 | CCCH-type zinc finger protein, control of rice leaf and tiller angle via BR signaling |
| 19 | Os06g0704400 | LOC_Os06g49090 | 29,744,334-29,750,632 | transposon protein |
| 20 | Os06g0704450 | None | 29,754,512-29,755,651 | hypothetical protein |
| 21 | Os06g0704500 | LOC_Os06g49100 | 29,754,932-29,756,377 | retrotransposon protein |
| 22 | Os06g0704600 | LOC_Os06g49110 | 29,757,543-29,761,819 | delta-aminolevulinic acid dehydratase, chloroplast precursor |
| 23 | Os06g0704700 | LOC_Os06g49120 | 29,762,530-29,766,745 | complex I intermediate-associated protein 30 domain containing protein |
| 24 | Os06g0704800 | LOC_Os06g49130 | 29,768,236-29,780,664 | TAZ zinc finger family protein |
| 25 | Os06g0704900 | LOC_Os06g49140 | 29,782,084-29,784,931 | ribosomal RNA large subunit methyltransferase J |
| 26 | Os06g0705000 | LOC_Os06g49150 | 29,786,520-29,788,667 | expressed protein |
| 27 | Os06g0705100 | LOC_Os06g49160 | 29,789,098-29,790,631 | thylakoid lumenal 16.5 kDa protein, chloroplast precursor |
| 28 | Os06g0705200 | LOC_Os06g49170 | 29,791,163-29,795,253 | OsWAK64 - OsWAK receptor-like protein kinase |
| 29 | Os06g0705250 | None | 29,791,592-29,795,228 | hypothetical gene |
| 30 | Os06g0705300 | LOC_Os06g49185 | 29,798,173-29,804,316 | plant-specific domain TIGR01589 family protein |
| 31 | Os06g0705350 | None | 29,804,661-29,807,002 | pentatricopeptide (PPR) repeat-containing protein |
| 32 | Os06g0705400 | LOC_Os06g49190 | 29,807,821-29,808,449 | lTPL154 - Protease inhibitor/seed storage/LTP family protein precursor |
| 33 | Os06g0705500 | LOC_Os06g49200 | 29,808,539-29,811,842 | ataxin-2, C-terminal domain containing protein |
| 34 | Os06g0705651 | None | 29,815,928-29,816,719 | hypothetical protein |
| 35 | Os06g0705700 | LOC_Os06g49220 | 29,824,393-29,826,498 | peptide transporter |
| 36 | Os06g0705901 | None | 29,824,636-29,826,498 | hypothetical protein |
| 37 | Os06g0706000 | None | 29,830,220-29,831,075 | hypothetical protein |
| 38 | Os06g0706100 | LOC_Os06g49240 | 29,828,537-29,831,137 | PTR-like peptide transporter |
| 39 | Os06g0706400 | LOC_Os06g49250 | 29,838,314-29,841,264 | nitrogen utilization efficiency, growth and grain yield |
| 40 | Os06g0706500 | LOC_Os06g49250 | 29,841,396-29,841,692 | peptide transporter PTR2 |
