# Supplementary material for: An Analysis of Natural Variation Reveals That OsFLA2 Controls Flag Leaf Angle in Rice (Oryza sativa L.)
Source: Front Plant Sci. 2022 Jun 23;13:906912. doi: 10.3389/fpls.2022.906912 (PMC9260283; doi:10.3389/fpls.2022.906912)
Supplement: Supplementary Table 7 — Candidate gene annotation in the region 2.27–2.47 Mb associated with FLA. [file Table_7.DOC]

**Table S7.** Candidate gene annotation in the region 2.27-2.47 Mb associated with flag leaf angle.

| Number | Gene ID | MSU ID | Position | Annotation |
| --- | --- | --- | --- | --- |
| 1 | Os02g0142060 | LOC_Os02g04915 | 2,274,553-2,275,429 | conserved hypothetical protein |
| 2 | Os02g0142100 | LOC_Os02g04924 | 2,277,044-2,279,844 | retrotransposon protein |
| 3 | Os02g0142250 | LOC_Os02g04945 | 2,312,299-2,317,211 | phosphatidate cytidylyltransferase family protein |
| 4 | Os02g0142300 | LOC_Os02g04950 | 2,318,214-2,323,337 | conserved hypothetical protein |
| 5 | Os02g0142400 | LOC_Os02g04960 | 2,325,414-2,328,080 | conserved hypothetical protein |
| 6 | Os02g0142450 | None | 2,331,669-2,345,472 | conserved hypothetical protein |
| 7 | Os02g0142500 | LOC_Os02g04970 | 2,343,538-2,345,234 | suppressor of auxin resistance 1 |
| 8 | Os02g0142750 | None | 2,368,844-2,370,387 | hypothetical gene |
| 9 | Os02g0142800 | LOC_Os02g05000 | 2,369,616-2,371,348 | expressed protein |
| 10 | Os02g0142850 | None | 2,372,150-2,373,061 | non-protein coding transcript. |
| 11 | Os02g0142875 | None | 2,372,240-2,374,416 | hypothetical protein |
| 12 | Os02g0142950 | None | 2,386,654-2,387,525 | suppressor of auxin resistance1 |
| 13 | Os02g0143100 | LOC_Os02g05030 | 2,388,400-2,394,345 | sucrose-phosphatase |
| 14 | Os02g0143200 | LOC_Os02g05040 | 2,397,461-2,404,964 | cyclin-related protein |
| 15 | Os02g0143300 | LOC_Os02g05050 | 2,406,632-2,407,019 | OsSAUR4 - Auxin-responsive SAUR gene family member |
| 16 | Os02g0143350 | None | 2,409,033-2,409,537 | auxin-responsive SAUR gene family member |
| 17 | Os02g0143400 | LOC_Os02g05060 | 2,408,927-2,409,319 | similar to H0114G12.9 protein |
| 18 | Os02g0144200 | LOC_Os02g05120 | 2,444,421-2,445,497 | expressed protein |
| 19 | Os02g0144300 | None | 2,447,941-2,451,093 | Similar to H0114G12.9 protein |
