# Supplementary material for: An Analysis of Natural Variation Reveals That OsFLA2 Controls Flag Leaf Angle in Rice (Oryza sativa L.)
Source: Front Plant Sci. 2022 Jun 23;13:906912. doi: 10.3389/fpls.2022.906912 (PMC9260283; doi:10.3389/fpls.2022.906912)
Supplement: Supplementary Table 8 — Base information of 30 Oryza rufipogon reported by Huang et al. (2012) (https://www.ebi.ac.uk/ena/browser/view/PRJEB2829). [file Table_8.DOC]

**Table S8.** Base information of 30 *Oryza rufipogon* reported by Huang et al. (2012) (https://www.ebi.ac.uk/ena/browser/view/PRJEB2829)

| Acc. No. | DRR No. | NIG Core collection rank | Species | Genome | Origin / Country |
| --- | --- | --- | --- | --- | --- |
| W0106 | DRR000348 | Rank1 | O. rufipogon | AA | India |
| W0120 | SRR1016477 | Rank1 | O. rufipogon | AA | India |
| W0137 | DRR226054 | Rank3 | O. rufipogon | AA | India |
| W0180 | DRR001188 | Rank3 | O. rufipogon | AA | Thailand |
| W0593 | SRR1016473 | Rank3 | O. rufipogon | AA | Malaya (Malaysia) |
| W0630 | SRR1016490 | Rank2 | O. rufipogon | AA | Burma |
| W1230 | DRR001189 | Rank3 | O. rufipogon | AA | Dutch New Guinea (Indonesia) |
| W1236 | DRR001184 | Rank2 | O. rufipogon | AA | Australian New Guinea (Papua New Guinea) |
| W1294 | SRR1016480 | Rank1 | O. rufipogon | AA | Philippines |
| W1551 | DRR226056 | Rank3 | O. rufipogon | AA | Thailand |
| W1669 | DRR226057 | Rank3 | O. rufipogon | AA | India |
| W1681 | DRR226058 | Rank3 | O. rufipogon | AA | India |
| W1715 | DRR226059 | Rank3 | O. rufipogon | AA | China |
| W1807 | SRR1016483 | Rank2 | O. rufipogon | AA | Sri Lanka |
| W1866 | DRR226061 | Rank1 | O. rufipogon | AA | Thailand |
| W1886 | SRR1016492 | no Rank | O. rufipogon | AA | Thailand |
| W1921 | DRR226062 | Rank1 | O. rufipogon | AA | Thailand |
| W1945 | DRR226063 | Rank2 | O. rufipogon | AA |  |
| W1962 | DRR226064 | no Rank | O. rufipogon | AA | China |
| W1965 | SRR1016475 | no Rank | O. rufipogon | AA | China |
| W1976 | SRR1016481 | no Rank | O. rufipogon | AA | Indonesia |
| W1981 | DRR001190 | Rank3 | O. rufipogon | AA | Indonesia |
| W2003 | SRR1016485 | Rank1 | O. rufipogon | AA | India |
| W2051 | DRR226066 | Rank2 | O. rufipogon | AA | Bangladesh |
| W2057 | SRR1016487 | no Rank | O. rufipogon | AA | Bangladesh |
| W2078 | DRR226068 | Rank2 | O. rufipogon | AA | Australia |
| W2109 | DRR226070 | Rank3 | O. rufipogon | AA | Australia |
| W2263 | DRR226071 | Rank2 | O. rufipogon | AA | Cambodia |
| W2114 | DRR058028 | no Rank | O. rufipogon | AA | Australia |
| W2117 | DRR058030 | no Rank | O. rufipogon | AA | Australia |

Reference:

Huang, X.H., Kurata, N., Wei, X.H., Wang, Z.X., Wang, A.H., Zhao, Q., et al. (2012). A map of rice genome variation reveals the origin of cultivated rice. *Nature* 490, 497–501. doi:10.1038/nature11532
