# Supplementary material for: An Analysis of Natural Variation Reveals That OsFLA2 Controls Flag Leaf Angle in Rice (Oryza sativa L.)
Source: Front Plant Sci. 2022 Jun 23;13:906912. doi: 10.3389/fpls.2022.906912 (PMC9260283; doi:10.3389/fpls.2022.906912)
Supplement: Supplementary Table 9 — Base information of 202 Oryza sativa downloaded from RiceVarMap version 2.0. [file Table_9.DOC]

**Table S9.** Base information of 202 Oryza sativa download from RiceVarMap v2.0

| Cultivar Name | ID Name | Subpopulation | Accession No. | Original/Country |
| --- | --- | --- | --- | --- |
| Zacaodao_13 | B049 | AUS | ERS470264 | Nepal |
| N_22 | C013 | AUS | SRR1239613 | Philippines |
| Dular | C015 | AUS | SRR1239615 | Philippines |
| KASALATH | CX227 | AUS | ERS470551 | Japan |
| ASWINA_330 | W330 | AUS | SRR1240133 | Bangladesh |
| Karnal_Local | W215 | AUS | SRR1240018 | India |
| DHAN_263 | IRIS-313-11025 | AUS | ERS469822 | Pakistan |
| JHONA_101 | IRIS-313-11027 | AUS | ERS469825 | Pakistan |
| MUSHKAN_340_A | IRIS-313-11029 | AUS | ERS469827 | Pakistan |
| NAWAN_SATHRA_BAGAR-344 | IRIS-313-11031 | AUS | ERS469829 | Pakistan |
| ARC_11751 | IRIS-313-10869 | AUS | ERS469672 | India |
| ARC_11777 | IRIS-313-10871 | AUS | ERS469675 | India |
| ARC_11822 | IRIS-313-10873 | AUS | ERS469677 | India |
| ARC_12021 | IRIS-313-10875 | AUS | ERS469679 | India |
| ARC_12067 | IRIS-313-10876 | AUS | ERS469680 | India |
| ARC_12101 | IRIS-313-10878 | AUS | ERS469682 | India |
| Malaihong | B010 | IND1 | ERS470228 | Malaysia |
| 71011 | B181 | IND1 | ERS470386 | Australia |
| Vietnam_zadao | B009 | INDI | ERS470227 | Vietnam |
| Kahamu | B015 | INDI | ERS470233 | Romania |
| Xianluosichi | B024 | INDI | ERS470241 | Thailand |
| IRAT_10 | B039 | INDI | ERS470256 | Ivory Coast |
| Nanjing_11 | B059 | INDI | ERS470273 | China |
| Aijiaonante | B060 | INDI | ERS470274 | China |
| Guangluai_4 | B061 | INDI | ERS470275 | China |
| Nantehao_ | B062 | INDI | ERS470276 | China |
| Funingzipigengzi | B067 | INDI | ERS470281 | China |
| Qiuqianbai_ | B072 | INDI | ERS470286 | China |
| Taishannuo | B074 | INDI | ERS470288 | China |
| Esiniu | B079 | INDI | ERS470292 | China |
| Heidu_4 | B081 | INDI | ERS470293 | China |
| Dongtingwanxian | B083 | INDI | ERS470295 | China |
| Gongju_73 | B092 | INDI | ERS470304 | China |
| Qitougu | B093 | INDI | ERS470305 | China |
| Zinuo | B094 | INDI | ERS470306 | China |
| Jiabala | B104 | INDI | ERS470314 | India |
| Xianggu | B108 | INDI | ERS470318 | China |
| Liusha_1 | B112 | INDI | ERS470322 | China |
| Chengduai_3 | B114 | INDI | ERS470324 | China |
| Aimakang | B115 | INDI | ERS470325 | China |
| Guangluai_15_1 | B119 | INDI | ERS470329 | China |
| Luke_3 | B121 | INDI | ERS470331 | China |
| Zaoxian_240 | B126 | INDI | ERS470336 | China |
| Dangyu_5_ | B127 | INDI | ERS470337 | China |
| Wanlixian | B130 | INDI | ERS470340 | China |
| Pei_C122 | B140 | INDI | ERS470350 | China |
| Baikehanhe | B147 | INDI | ERS470357 | China |
| Haoxiang | B149 | INDI | ERS470359 | China |
| Jiefangxian | B198 | INDI | ERS470402 | China |
| Lucaihao | B208 | INDI | ERS470411 | China |
| Sadu_cho | C001 | INDI | SRR1239601 | Philippines |
| Aijiaonante | C019 | INDI | SRR1239619 | China |
| TN1 | CX162 | INDI | ERS470527 | China |
| CHIAY1-WU-K'O | IRIS-313-11692 | INDI | ERS468867 | China |
| MR84 | CX156 | INDII | ERS470521 | Malaysia |
| Dumai | B029 | INDII | ERS470246 | India |
| PRohini | B032 | INDII | ERS470249 | India |
| BW_293-2 | B033 | INDII | ERS470250 | Sri lanka |
| Erjiunan_1 | B058 | INDII | ERS470272 | China |
| IR661-1 | B139 | INDII | ERS470349 | China |
| JWR_221 | B146 | INDII | ERS470356 | China |
| ECIA_179-S13 | B194 | INDII | ERS470398 | Cuba |
| PMS_10B | B195 | INDII | ERS470399 | India |
| Jinyou_1 | B200 | INDII | ERS470404 | China |
| MILYANG_23 | C011 | INDII | SRR1239611 | Taiwan |
| Y134 | CX15 | INDII | ERS470514 | China |
| Chorofa | CX150 | INDII | ERS470515 | Philippines |
| IR42 | CX161 | INDII | ERS470526 | Philippines |
| IR2061-522-6-9 | CX206 | INDII | ERS470536 | Philippines |
| Bg90-2 | CX21 | INDII | ERS470538 | Sri lanka |
| Types3 | CX218 | INDII | ERS470544 | India |
| PR106 | CX22 | INDII | ERS470546 | India |
| IR77298-14-1-2 | CX225 | INDII | ERS470549 | Philippines |
| IR06G113 | CX226 | INDII | ERS470550 | Philippines |
| IR64-IL | CX230 | INDII | ERS470554 | China |
| IR62266-42-6-2 | CX234 | INDII | ERS470558 | Philippines |
| Amo13_(Sana) | W172 | INDII | SRR1239975 | Philippines |
| Gayabyeo | W174 | INDII | SRR1239977 | Korea |
| Govnd | W237 | INDII | SRR1240040 | Philippines |
| Pokhreli | W236 | INDII | SRR1240039 | Philippines |
| PR106-1 | W170 | INDII | SRR1239973 | Pakistan |
| X23 | W231 | INDII | SRR1240034 | Vietnam |
| WAGASAN(RED) | IRIS-313-11511 | INDII | ERS468720 | Philippines |
| ADIALLO | IRIS-313-11523 | INDII | ERS468724 | Senegal |
| IRAT144 | CX247 | INDII | ERS470568 | Indonesia |
| IR68897B | CX281 | INDII | ERS470592 | Philippines |
| LX2007 | CX303 | INDII | ERS470605 | China |
| ZH5 | CX305 | INDII | ERS470607 | China |
| Hnankar | CX31 | INDII | ERS470610 | Myanmar |
| R106 | CX313 | INDII | ERS470611 | China |
| Tianhanhao | B007 | INDIII | ERS470225 | Vietnam |
| C_894_21 | B027 | INDIII | ERS470244 | Philippines |
| Jaibattey | B030 | INDIII | ERS470247 | India |
| Ngatsin | B031 | INDIII | ERS470248 | India |
| Zhongnong_4 | B087 | INDIII | ERS470299 | China |
| Mowangguneiza | B095 | INDIII | ERS470307 | China |
| Sri_Raja | CX148 | INDIII | ERS470512 | Malaysia |
| Jalmagna | CX153 | INDIII | ERS470518 | India |
| Khao_Dawk_Mali_105 | CX154 | INDIII | ERS470519 | Thailand |
| Madhukar | CX155 | INDIII | ERS470520 | India |
| Shwe_War_Tun | CX158 | INDIII | ERS470523 | Vietnam |
| Rasi | CX23 | INDIII | ERS470553 | Indonesia |
| ARC_18001 | IRIS-313-11284 | INDIII | ERS470072 | India |
| ARC_18064 | IRIS-313-11285 | INDIII | ERS470074 | India |
| ARC_18533 | IRIS-313-11443 | INDIII | ERS468660 | India |
| Laozaogu | B246 | INDIII | ERS470444 | China |
| ANENOE | IRIS-313-11495 | INDIII | ERS468706 | Indonesia |
| PADI_UDANG | IRIS-313-11499 | INDIII | ERS468711 | Indonesia |
| HOE_PHAR_THAE | IRIS-313-11541 | INDIII | ERS468739 | Myanmar |
| MOE_GAUNG_PYU | IRIS-313-11545 | INDIII | ERS468744 | Myanmar |
| SHWE_SABA | IRIS-313-11547 | INDIII | ERS468746 | Myanmar |
| PA_WOON | IRIS-313-11555 | INDIII | ERS468752 | Sierra Leone |
| Heibiao | B001 | TEJ | ERS470219 | China |
| Sansuijin | B002 | TEJ | ERS470220 | China |
| Qiuguangtengxi | B004 | TEJ | ERS470222 | Japan |
| Wanshi | B005 | TEJ | ERS470223 | Japan |
| Baxiang | B008 | TEJ | ERS470226 | Vietnam |
| Wuziluosi | B014 | TEJ | ERS470232 | Soviet union |
| Aomierte_168 | B016 | TEJ | ERS470234 | Hungary |
| Aerjituo | B017 | TEJ | ERS470235 | Bulgaria |
| Albania_Rice | B034 | TEJ | ERS470251 | Albania |
| Nabated_A_Smar | B038 | TEJ | ERS470255 | Egypt |
| Gongchengxiang | B045 | TEJ | ERS470260 | Japan |
| Qiutianxiaoting | B046 | TEJ | ERS470261 | Japan |
| Zhenfu_8 | B047 | TEJ | ERS470262 | Japan |
| YR196 | B055 | TEJ | ERS470269 | Australia |
| Tieganwu_ | B056 | TEJ | ERS470270 | China |
| Xiushui_115 | B057 | TEJ | ERS470271 | China |
| Guangkexiangnuo | B066 | TEJ | ERS470280 | China |
| Gaoyangdiandao_dahongmang | B068 | TEJ | ERS470282 | China |
| Muxiqiu | B071 | TEJ | ERS470285 | China |
| Yizhixiang | B077 | TEJ | ERS470291 | China |
| Hongkezhenuo | B100 | TEJ | ERS470310 | China |
| Yangkenuo | B101 | TEJ | ERS470311 | China |
| Maguzi | B102 | TEJ | ERS470312 | China |
| Laohongdao | B103 | TEJ | ERS470313 | China |
| Lengshuigu_2 | B109 | TEJ | ERS470319 | China |
| Huangpinuo | B110 | TEJ | ERS470320 | China |
| Zimangfeie | B111 | TEJ | ERS470321 | China |
| Lixingeng | B117 | TEJ | ERS470327 | China |
| Liaogeng_287 | B122 | TEJ | ERS470332 | China |
| Geng_87-304 | B124 | TEJ | ERS470334 | China |
| Yuyannuo | B136 | TEJ | ERS470346 | China |
| Zaoshunonghu-6 | B152 | TEJ | ERS470362 | China |
| LimingB | B154 | TEJ | ERS470364 | China |
| Shuiyuan_300Li_ | B160 | TEJ | ERS470370 | China |
| Jianghuadao_ | B166 | TEJ | ERS470376 | China |
| Qinglinzaosheng | B167 | TEJ | ERS470377 | China |
| Chimao | B182 | TEJ | ERS470387 | Japan |
| Longhuamaohu | B204 | TEJ | ERS470408 | China |
| Cunsanli | B205 | TEJ | ERS470409 | China |
| Chikenuo | B212 | TEJ | ERS470413 | China |
| Tainong67 | C012 | TEJ | SRR1239612 | Philippines |
| Laoguangtou83 | C016 | TEJ | SRR1239616 | China |
| Xingguo | C171 | TEJ | SRR1239771 | China |
| Zhonghua8hao | C172 | TEJ | SRR1239772 | China |
| Yuan_jing_7 | CX16 | TEJ | ERS470524 | China |
| Wuyugeng_3_ | CX165 | TEJ | ERS470528 | China |
| Qb_604 | CX210 | TEJ | ERS470539 | China |
| M3122 | CX211 | TEJ | ERS470540 | China |
| Nongken_58 | CX212 | TEJ | ERS470541 | Japan |
| Chennong_89366 | CX213 | TEJ | ERS470542 | China |
| C71 | W232 | TEJ | SRR1240035 | Vietnam |
| Guantuibaihe | W325 | TEJ | SRR1240128 | China |
| Dahonggu | HP147 | TEJ | ERR037175 | China |
| Xiaominian | HP145 | TEJ | ERR037173 | China |
| AR_133 | IRIS-313-11493 | TEJ | ERS468704 | India |
| 91-382 | IRIS-313-11661 | TEJ | ERS468838 | Bhutan |
| CN1067 | IRIS-313-11702 | TEJ | ERS468901 | United States |
| Han_502 | CX284 | TEJ | ERS470594 | China |
| C52 | CX287 | TEJ | ERS470597 | China |
| HP121 | CX307 | TEJ | ERS470609 | China |
| Huangkedao | B018 | TRJ | ERS470236 | United States |
| Djanda_Mangja | B025 | TRJ | ERS470242 | Indonesia |
| Nanoay_P.A | B037 | TRJ | ERS470254 | Argentina |
| YR_83-23-11 | B043 | TRJ | ERS470258 | Australlia |
| 80A97YR303-304-1-3 | B053 | TRJ | ERS470267 | Australia |
| 80050YR72136-43 | B054 | TRJ | ERS470268 | Australia |
| IRAT_36 | B188 | TRJ | ERS470392 | Ivory Coast |
| IRAT_669 | B189 | TRJ | ERS470393 | Ivory Coast |
| ITA_221 | B190 | TRJ | ERS470394 | Nigeria |
| Taidongludao | B196 | TRJ | ERS470400 | China |
| AzuCENA | C005 | TRJ | SRR1239605 | Philippines |
| CYPRESs | C010 | TRJ | SRR1239610 | Philippines |
| AZUCENA | CX151 | TRJ | ERS470516 | Philippines |
| Brazil_Nuodao | CX214 | TRJ | ERS470543 | Brazil |
| IRAT109 | CX220 | TRJ | ERS470547 | Brazil |
| Khazar | W173 | TRJ | SRR1239976 | Iran |
| GPNO_5055 | W329 | TRJ | SRR1240132 | United States |
| PADI_RENDAH | IRIS-313-11313 | TRJ | ERS470104 | Indonesia |
| PELAU | IRIS-313-11314 | TRJ | ERS470105 | Indonesia |
| ARC_11946 | IRIS-313-10874 | TRJ | ERS469678 | India |
| BALU_PR1A | IRIS-313-11496 | TRJ | ERS468707 | Indonesia |
| PADI_ARA | IRIS-313-11497 | TRJ | ERS468708 | Indonesia |
| TV30 | IRIS-313-11507 | TRJ | ERS468715 | Vietnam |
| CHATO | IRIS-313-11513 | TRJ | ERS468722 | Ecuador |
| KOUlNONEPOU | IRIS-313-11524 | TRJ | ERS468725 | Ivory Coast |
| GOLO | IRIS-313-11527 | TRJ | ERS468728 | Ivory Coast |
| MARIA_GAKIT | IRIS-313-11532 | TRJ | ERS468731 | Philippines |
| PATIE_ROUGE | IRIS-313-11540 | TRJ | ERS468738 | Guinea |
| IAC47 | CX241 | TRJ | ERS470565 | Philippines |
| Naravilha | CX280 | TRJ | ERS470591 | Brazil |
