# Supplementary material for: An Analysis of Natural Variation Reveals That OsFLA2 Controls Flag Leaf Angle in Rice (Oryza sativa L.)
Source: Front Plant Sci. 2022 Jun 23;13:906912. doi: 10.3389/fpls.2022.906912 (PMC9260283; doi:10.3389/fpls.2022.906912)
Supplement: Supplementary Table 10 — Single nucleotide polymorphism distribution of OsFLA6 and OsFLA2 in 30 O. rufipogon. [file Table_10.DOC]

**Table S10.** SNP distribution of *OsFLA6* and *OsFLA2* in 30 Oryza rufipogon

| Acc. No. | SNP distribution | | | | | | | | | |
| --- | --- | --- | --- | --- | --- | --- | --- | --- | --- | --- |
|  | *OsFLA6_Os06g0704300* | | |  | *OsFLA2_Os02g0142875* | | | | | |
|  | 29739644 | 29739976 | 29740769 |  | 2372278 | 2372437 | 2372667 | 2374358 | 2374376 | 2374409 |
| W0106 | G | G | A |  | T | C | G | A | A | G |
| W0120 | G | G | G |  | C | C | G | A | A | G |
| W0137 | G | G | G |  | T | C | G | A | G | A |
| W0180 | G | G | A |  | T | C | G | A | G | A |
| W0593 | G | G | G |  | T | C | G | A | G | A |
| W0630 | G | G | A |  | T | C | G | A | G | G |
| W1230 | G | G | A |  | T | C | G | A | A | G |
| W1236 | G | G | G |  | C | C | G | A | G | A |
| W1294 | G | G | G |  | C | C | G | A | G | A |
| W1551 | G | G | A |  | T | C | G | A | G | A |
| W1669 | G | A | G |  | T | C | G | A | G | A |
| W1681 | G | G | A |  | T | C | G | A | A | G |
| W1715 | A | A | G |  | T | C | G | T | A | G |
| W1807 | A | G | G |  | T | C | G | T | A | G |
| W1866 | G | G | A |  | T | C | G | A | A | G |
| W1886 | A | G | A |  | T | T | G | T | G | A |
| W1921 | A | G | A |  | T | C | G | A | A | G |
| W1945 | A | A | G |  | T | C | G | A | G | A |
| W1962 | G | A | G |  | C | T | G | T | A | G |
| W1965 | A | A | G |  | C | T | G | T | A | G |
| W1976 | G | G | G |  | C | T | G | T | A | G |
| W1981 | G | G | A |  | T | C | G | A | G | G |
| W2003 | A | G | G |  | T | C | G | A | G | A |
| W2051 | A | G | G |  | T | C | G | A | G | G |
| W2057 | A | G | A |  | T | C | G | A | G | A |
| W2078 | G | G | G |  | T | C | G | A | G | A |
| W2109 | A | G | A |  | C | T | G | T | A | G |
| W2263 | A | G | A |  | T | C | G | T | A | G |
| W2114 | A | A | G |  | C | T | G | T | A | G |
| W2117 | A | G | A |  | C | T | G | T | A | G |
