# Supplementary material for: An Analysis of Natural Variation Reveals That OsFLA2 Controls Flag Leaf Angle in Rice (Oryza sativa L.)
Source: Front Plant Sci. 2022 Jun 23;13:906912. doi: 10.3389/fpls.2022.906912 (PMC9260283; doi:10.3389/fpls.2022.906912)
Supplement: Supplementary Table 11 — Single nucleotide polymorphism distribution of OsFLA6 and OsFLA2 in 202 O. sativa. [file Table_11.DOC]

**Table S11.** SNP distribution of *OsFLA6* and *OsFLA2* in 202 *Oryza sativa*.

| ID Name | SNP distribution | | | | | | | | | |
| --- | --- | --- | --- | --- | --- | --- | --- | --- | --- | --- |
|  | *OsFLA6_Os06g0704300* | | |  | *OsFLA2_Os02g0142875* | | | | | |
|  | 29739644 | 29739976 | 29740769 |  | 2372278 | 2372437 | 2372667 | 2374358 | 2374376 | 2374409 |
| B049 | G | G | A |  | T | C | G | A | A | G |
| C013 | G | G | A |  | T | C | G | A | A | G |
| C015 | G | G | A |  | T | T | T | A | G | A |
| CX227 | G | G | A |  | T | C | G | A | A | G |
| W330 | G | G | A |  | T | C | G | A | A | G |
| W215 | G | G | A |  | T | C | G | A | A | G |
| IRIS-313-11025 | G | G | A |  | T | C | G | A | A | G |
| IRIS-313-11027 | G | G | A |  | T | C | G | A | A | G |
| IRIS-313-11029 | G | G | A |  | T | C | G | A | A | G |
| IRIS-313-11031 | G | G | A |  | T | C | G | A | A | G |
| IRIS-313-10869 | G | G | A |  | T | C | G | A | A | G |
| IRIS-313-10871 | G | G | A |  | T | C | G | A | A | G |
| IRIS-313-10873 | G | G | A |  | T | C | G | A | A | G |
| IRIS-313-10875 | G | G | A |  | T | C | G | A | A | G |
| IRIS-313-10876 | G | G | A |  | T | T | T | A | G | A |
| IRIS-313-10878 | G | G | A |  | T | C | G | A | A | G |
| B010 | G | G | A |  | T | C | G | T | A | G |
| B181 | G | G | A |  | T | C | G | T | A | G |
| B009 | G | G | A |  | C | C | G | A | G | A |
| B015 | G | G | A |  | T | C | G | T | A | G |
| B024 | G | G | A |  | T | C | G | A | A | G |
| B039 | G | G | A |  | T | C | G | T | A | G |
| B059 | A | G | A |  | T | C | G | T | A | G |
| B060 | A | G | A |  | T | C | G | T | A | G |
| B061 | A | G | A |  | C | C | G | A | G | A |
| B062 | A | G | A |  | T | C | G | T | A | G |
| B067 | A | G | A |  | T | C | G | A | A | G |
| B072 | G | G | A |  | T | C | G | A | A | G |
| B074 | G | G | A |  | T | C | G | T | A | G |
| B079 | G | G | A |  | T | C | G | T | A | G |
| B081 | G | G | A |  | C | C | G | A | G | A |
| B083 | G | G | A |  | C | T | T | A | G | G |
| B092 | G | G | A |  | T | C | G | T | A | G |
| B093 | G | G | A |  | C | C | G | A | G | A |
| B094 | G | G | A |  | T | C | G | T | A | G |
| B104 | G | G | A |  | C | C | G | A | G | A |
| B108 | G | G | A |  | C | T | T | A | A | G |
| B112 | G | G | A |  | T | C | G | A | G | A |
| B114 | G | G | A |  | T | C | G | T | A | G |
| B115 | G | G | A |  | T | C | G | T | A | G |
| B119 | G | G | A |  | T | C | G | T | A | G |
| B121 | G | G | A |  | T | C | G | T | A | G |
| B126 | G | G | A |  | T | C | G | T | A | G |
| B127 | G | G | A |  | T | C | G | T | A | G |
| B130 | G | G | A |  | C | C | G | A | G | A |
| B140 | G | G | A |  | C | C | G | A | G | A |
| B147 | G | G | A |  | T | C | G | T | A | G |
| B149 | G | G | A |  | T | C | G | T | A | G |
| B198 | G | G | A |  | T | C | G | A | A | G |
| B208 | G | G | A |  | C | C | G | A | G | A |
| C001 | G | G | A |  | T | T | T | A | G | A |
| C019 | G | G | A |  | T | C | G | T | A | G |
| CX162 | G | G | A |  | T | C | G | T | A | G |
| IRIS-313-11692 | G | G | A |  | T | C | G | T | A | G |
| CX156 | G | G | A |  | C | T | G | A | G | G |
| B029 | G | G | A |  | T | C | G | T | A | G |
| B032 | G | G | A |  | C | C | G | A | G | A |
| B033 | G | G | A |  | T | C | G | T | A | G |
| B058 | A | G | A |  | T | C | G | T | A | G |
| B139 | G | G | A |  | C | C | G | A | G | G |
| B146 | G | G | A |  | T | C | G | T | A | G |
| B194 | G | G | A |  | T | C | G | A | G | A |
| B195 | G | A | A |  | T | C | G | A | A | G |
| B200 | G | G | A |  | T | C | G | A | G | G |
| C011 | G | G | A |  | T | C | G | T | A | G |
| CX15 | G | G | A |  | C | T | G | A | G | G |
| CX150 | G | G | A |  | C | C | G | A | G | A |
| CX161 | G | A | G |  | T | C | G | T | A | G |
| CX206 | G | A | G |  | C | T | G | A | G | G |
| CX21 | G | G | A |  | C | T | T | A | G | G |
| CX218 | G | G | A |  | T | C | G | T | A | G |
| CX22 | G | G | A |  | T | C | G | T | A | G |
| CX225 | G | G | A |  | T | C | G | T | A | G |
| CX226 | G | G | A |  | T | C | G | T | A | G |
| CX230 | G | G | A |  | T | C | G | T | A | G |
| CX234 | G | G | A |  | T | C | G | T | A | G |
| W172 | G | G | A |  | T | C | G | A | G | A |
| W174 | G | G | A |  | T | C | G | T | A | G |
| W237 | G | G | A |  | T | C | G | T | A | G |
| W236 | G | G | A |  | C | C | G | A | G | A |
| W170 | G | G | A |  | T | C | G | T | A | G |
| W231 | G | G | A |  | T | C | G | T | A | G |
| IRIS-313-11511 | G | G | A |  | T | T | T | A | G | A |
| IRIS-313-11523 | G | G | A |  | T | C | G | A | A | G |
| CX247 | G | A | G |  | C | C | G | A | G | A |
| CX281 | G | G | A |  | T | C | G | A | A | G |
| CX303 | G | G | A |  | C | C | G | A | G | A |
| CX305 | G | G | A |  | T | C | G | T | A | G |
| CX31 | G | G | A |  | C | T | T | A | G | G |
| CX313 | G | G | A |  | C | C | G | A | G | A |
| B007 | G | G | A |  | T | C | G | T | A | G |
| B027 | G | G | A |  | C | C | G | A | G | A |
| B030 | G | G | A |  | T | C | G | A | A | G |
| B031 | G | G | A |  | T | C | G | A | A | G |
| B087 | G | G | A |  | T | C | G | A | A | G |
| B095 | G | G | A |  | T | C | G | A | G | G |
| CX148 | G | G | A |  | C | T | G | A | G | G |
| CX153 | G | G | A |  | T | C | G | A | A | G |
| CX154 | G | G | A |  | C | C | G | A | G | G |
| CX155 | G | G | A |  | T | C | G | A | A | G |
| CX158 | G | G | A |  | C | C | G | A | G | A |
| CX23 | G | G | A |  | C | T | T | A | G | G |
| IRIS-313-11284 | G | G | A |  | T | C | G | A | A | G |
| IRIS-313-11285 | G | G | A |  | C | C | G | A | G | A |
| IRIS-313-11443 | G | G | A |  | C | T | G | A | G | G |
| B246 | G | G | A |  | T | C | G | A | G | G |
| IRIS-313-11495 | G | G | A |  | T | T | T | A | G | A |
| IRIS-313-11499 | G | G | A |  | T | T | T | A | G | A |
| IRIS-313-11541 | G | G | A |  | C | T | T | A | G | G |
| IRIS-313-11545 | G | G | A |  | C | T | G | A | G | G |
| IRIS-313-11547 | G | G | A |  | C | C | G | A | G | A |
| IRIS-313-11555 | G | G | A |  | C | C | G | A | G | A |
| B001 | A | A | G |  | T | C | G | A | G | A |
| B002 | A | A | G |  | T | C | G | A | G | A |
| B004 | A | A | G |  | T | C | G | A | G | A |
| B005 | A | A | G |  | T | C | G | A | G | A |
| B008 | A | A | G |  | T | C | G | A | G | A |
| B014 | G | A | G |  | T | C | G | A | G | A |
| B016 | A | A | G |  | T | C | G | A | G | A |
| B017 | A | A | G |  | T | C | G | A | G | A |
| B034 | A | A | G |  | T | C | G | A | G | A |
| B038 | G | A | G |  | T | C | G | A | G | A |
| B045 | A | A | G |  | T | C | G | A | G | A |
| B046 | A | A | G |  | T | C | G | A | G | A |
| B047 | A | A | G |  | T | C | G | A | G | A |
| B055 | A | A | G |  | T | C | G | A | G | A |
| B056 | A | A | G |  | T | C | G | A | G | A |
| B057 | A | A | G |  | T | C | G | A | G | A |
| B066 | A | A | G |  | T | T | T | A | G | A |
| B068 | A | A | G |  | T | C | G | A | G | A |
| B071 | A | A | G |  | T | C | G | A | G | A |
| B077 | A | A | G |  | T | C | G | A | G | A |
| B100 | A | A | G |  | T | C | G | A | G | A |
| B101 | A | A | G |  | T | C | G | A | G | A |
| B102 | G | A | G |  | T | T | T | A | G | A |
| B103 | A | A | G |  | T | C | G | A | G | A |
| B109 | G | A | G |  | T | C | G | A | G | A |
| B110 | A | A | G |  | T | C | G | A | G | A |
| B111 | A | A | G |  | T | C | G | A | G | A |
| B117 | A | A | G |  | T | C | G | A | G | A |
| B122 | A | A | G |  | T | C | G | A | G | A |
| B124 | A | A | G |  | T | C | G | A | G | A |
| B136 | A | A | G |  | T | C | G | A | G | A |
| B152 | A | A | G |  | T | C | G | A | G | A |
| B154 | A | A | G |  | T | C | G | A | G | A |
| B160 | A | A | G |  | T | C | G | A | G | A |
| B166 | A | A | G |  | T | C | G | A | G | A |
| B167 | A | A | G |  | T | T | T | A | G | A |
| B182 | A | A | G |  | T | T | T | A | G | A |
| B204 | A | A | G |  | T | C | G | A | G | A |
| B205 | A | A | G |  | T | C | G | A | G | A |
| B212 | A | A | G |  | T | C | G | A | G | A |
| C012 | A | A | G |  | T | C | G | A | G | A |
| C016 | A | A | G |  | T | C | G | A | G | A |
| C171 | A | A | G |  | T | C | G | A | G | A |
| C172 | A | A | G |  | T | C | G | A | G | A |
| CX16 | A | A | G |  | T | C | G | A | G | A |
| CX165 | A | A | G |  | T | C | G | A | G | A |
| CX210 | G | G | A |  | T | C | G | A | G | A |
| CX211 | A | A | G |  | T | C | G | A | G | A |
| CX212 | A | A | G |  | T | C | G | A | G | A |
| CX213 | A | A | G |  | T | C | G | A | G | A |
| W232 | G | A | G |  | T | C | G | A | A | G |
| W325 | G | A | G |  | T | C | G | A | G | A |
| HP147 | A | A | G |  | T | C | G | A | G | A |
| HP145 | A | A | G |  | T | C | G | A | G | A |
| IRIS-313-11493 | G | G | A |  | C | C | G | A | G | A |
| IRIS-313-11661 | A | A | G |  | T | C | G | A | G | A |
| IRIS-313-11702 | A | A | G |  | T | T | T | A | G | A |
| CX284 | G | G | A |  | T | C | G | A | G | A |
| CX287 | G | A | G |  | T | C | G | A | G | A |
| CX307 | G | A | G |  | T | C | G | A | G | A |
| B018 | G | G | A |  | T | C | G | A | G | A |
| B025 | G | G | A |  | T | T | T | A | G | A |
| B037 | G | A | G |  | T | T | T | A | G | A |
| B043 | G | A | G |  | T | T | T | A | G | A |
| B053 | A | A | G |  | T | C | G | A | G | A |
| B054 | A | A | G |  | T | C | G | A | G | A |
| B188 | G | A | G |  | T | T | T | A | G | A |
| B189 | G | G | A |  | T | T | T | A | G | A |
| B190 | G | A | G |  | T | T | T | A | G | A |
| B196 | G | G | A |  | T | C | G | A | G | A |
| C005 | G | G | A |  | T | T | T | A | G | A |
| C010 | G | G | A |  | T | C | G | A | G | A |
| CX151 | G | G | A |  | T | T | T | A | G | A |
| CX214 | G | A | G |  | T | T | T | A | G | A |
| CX220 | G | A | G |  | T | T | T | A | G | A |
| W173 | G | G | A |  | T | T | T | A | G | A |
| W329 | A | A | G |  | T | C | G | T | A | G |
| IRIS-313-11313 | G | G | A |  | T | T | T | A | G | A |
| IRIS-313-11314 | G | A | G |  | T | T | T | A | G | A |
| IRIS-313-10874 | G | G | A |  | T | T | T | A | G | A |
| IRIS-313-11496 | G | G | A |  | T | T | T | A | G | A |
| IRIS-313-11497 | G | G | A |  | C | C | G | A | G | A |
| IRIS-313-11507 | G | G | A |  | T | C | G | T | A | G |
| IRIS-313-11513 | G | G | A |  | C | T | T | A | G | G |
| IRIS-313-11524 | G | G | A |  | T | T | T | A | G | A |
| IRIS-313-11527 | G | A | G |  | T | T | T | A | G | A |
| IRIS-313-11532 | G | G | A |  | T | T | T | A | G | A |
| IRIS-313-11540 | G | G | A |  | T | T | T | A | G | A |
| CX241 | G | A | G |  | T | T | T | A | G | A |
| CX280 | G | G | A |  | T | C | G | A | G | A |
