# Supplementary material for: An Analysis of Natural Variation Reveals That OsFLA2 Controls Flag Leaf Angle in Rice (Oryza sativa L.)
Source: Front Plant Sci. 2022 Jun 23;13:906912. doi: 10.3389/fpls.2022.906912 (PMC9260283; doi:10.3389/fpls.2022.906912)
Supplement: Supplementary Table 12 — The results of QTLs for FLA detected in this study overlapped with the QTLs/genes of rice FLA reported previously. [file Table_12.DOC]

**Table S12.** The results of QTLs for FLA detected in this study overlapped with the QTLs/Genes of rice flag leaf angle reported previously.

| QTL name | Chr. | Position range | Known gene^a^ | Known QTL^a^/  association locus^a^ | Flanking region^a^ | Reference |
| --- | --- | --- | --- | --- | --- | --- |
| *qFLA1* | 1 | 31458572-31658572 |  | *qFLA1* | 27034336-42953262 | Dong et al. 2003 |
| *qFLA4* | 4 | 4551577-4751577 |  | *qFLA4* | 1350218-5375422  177080-19463179 | Zhang et al. 2013  Ham et al. 2019 |
| *qFLA5* | 5 | 22233371-22433371 |  | *qFLA5-1* | 14511624-29285186 | Li et al. 1999 |
| *qFLA6* | 6 | 29639644-29839644 | *OsLIC1* |  | 29738880-29742499 | Wang et al. 2008  Zhang et al. 2012 |
| *qFLA9* | 9 | 14828244-15028244 |  | *qFLA9* | 2351092-24585902 | Li et al. 1999 |

^a^The gene name, QTL name and the physical position (bp) was inferred the from the database of Gramene website (http://www.gramene.org/markers/), BLAST (http://blast.ncbi.nlm.nih.gov/Blast.cgi) and the China Rice Data Center database (http://www.ricedata.cn/gene/list/1499.htm).
